# Lower Muscarinic M_1_ Receptor Availability in Schizophrenia: In Vivo Positron Emission Tomography Evidence

**DOI:** 10.1016/j.biopsych.2026.06.002

**Published:** 2026-06-12

**Authors:** Tommaso Volpi, Rajiv Radhakrishnan, Rachel Hird, Mika Naganawa, Nabeel Nabulsi, Soheila Najafzadeh, Swanee Jacutin-Porte, David Labaree, Richard E. Carson, Yiyun Huang, Deepak C. D’Souza

**Affiliations:** Department of Radiology and Biomedical Engineering, Yale University School of Medicine, New Haven, Connecticut; Department of Radiology and Biomedical Engineering, Yale University School of Medicine, New Haven, Connecticut; Department of Psychiatry, Yale University School of Medicine, New Haven, Connecticut; Psychiatry Service, Veterans Affairs Connecticut Healthcare System, West Haven, Connecticut; Abraham Ribicoff Research Facilities, Connecticut Mental Health Center, New Haven, Connecticut; Department of Psychiatry, Yale University School of Medicine, New Haven, Connecticut; Abraham Ribicoff Research Facilities, Connecticut Mental Health Center, New Haven, Connecticut; Department of Radiology and Biomedical Engineering, Yale University School of Medicine, New Haven, Connecticut; Department of Radiology and Biomedical Engineering, Yale University School of Medicine, New Haven, Connecticut; Department of Radiology and Biomedical Engineering, Yale University School of Medicine, New Haven, Connecticut; Department of Radiology and Biomedical Engineering, Yale University School of Medicine, New Haven, Connecticut; Department of Radiology and Biomedical Engineering, Yale University School of Medicine, New Haven, Connecticut; Department of Radiology and Biomedical Engineering, Yale University School of Medicine, New Haven, Connecticut; Department of Radiology and Biomedical Engineering, Yale University School of Medicine, New Haven, Connecticut; Department of Psychiatry, Yale University School of Medicine, New Haven, Connecticut; Psychiatry Service, Veterans Affairs Connecticut Healthcare System, West Haven, Connecticut; Abraham Ribicoff Research Facilities, Connecticut Mental Health Center, New Haven, Connecticut

## Abstract

**BACKGROUND:**

There is growing interest in the contributions of brain muscarinic M_1_ receptors to the neurobiology of schizophrenia (SZ). Postmortem evidence strongly indicates M_1_ deficits in patients with SZ. Recently, the U.S. Food and Drug Administration approved xanomeline and trospium chloride, which contains the M_1_/M_4_ agonist xanomeline, as the first nondopaminergic medication for SZ. The development of the positron emission tomography ligand ^11^C-LSN3172176 enables in vivo quantification of brain M_1_ availability in SZ and its relationship to clinical features.

**METHODS:**

M_1_ availability in patients with SZ (*n* = 16) was compared with age- and sex-matched healthy control (HC) participants (*n* = 16) using ^11^C-LSN3172176 and high-resolution research tomography. Distribution volume ratio relative to the centrum semiovale (*DVR*_CS_) and distribution volume (*V*_T_) were measured. Regional variation in percent gray matter fraction was included as a covariate to account for potential atrophy.

**RESULTS:**

Compared with HCs, patients with SZ showed significantly lower M_1_ availability across several cortical and subcortical regions, with large effect sizes: frontal (*DVR*_CS_: −13%), temporal (*DVR*_CS_: −15%, *V*_T_: −12%), parietal (*DVR*_CS_: −14%, *V*_T_: −11%), and occipital (*DVR*_CS_: −16%, *V*_T_: −13%) cortices; caudate (*DVR*_CS_: −19%, *V*_T_: −15%); putamen (*DVR*_CS_: −19%, *V*_T_: −17%); hippocampus (*DVR*_CS_: −13%); and amygdala (*DVR*_CS_: −19%, *V*_T_: −16%). A subgroup of patients (*DVR*_CS_: 44%, *V*_T_: 27%) showed larger whole-brain M_1_ deficits (>20% from the mean of HCs). Exploratory analyses suggested associations between M_1_ availability and selected clinical measures.

**CONCLUSIONS:**

Together with postmortem data, these in vivo findings warrant further characterization of M_1_ deficits in SZ and highlight M_1_ as a therapeutic target and potential biomarker for muscarinic-based treatments.

Schizophrenia (SZ) is a serious mental disorder that is heterogeneous in its expression and biology (1). Beyond dopamine, many other neurotransmitter systems have been implicated in its pathophysiology, including glutamate, GABA (gamma-aminobutyric acid), and acetylcholine (ACh) (2).

The pharmacological treatment of SZ has been dominated by dopamine D_2_ receptor antagonist/agonists (3). Their limited efficacy, especially for negative and cognitive symptoms, and their significant side effects have spurred the search for drugs with alternative mechanisms of action.

Recently, the U.S. Food and Drug Administration (FDA) approved xanomeline-trospium (KarXT) as the first nondopaminergic medication for the treatment of SZ (4). Xanomeline is a partial agonist of muscarinic M_1_/M_4_ receptors, and trospium chloride is a peripheral anticholinergic. M_1_ receptors are G protein–coupled receptors that are present throughout the cortex and subcortical regions. Converging lines of evidence from postmortem and preclinical studies provide strong support for the hypothesis that brain muscarinic M_1_ receptor deficits are present in SZ (5–8), with some authors suggesting that a subset of patients with SZ has a muscarinic receptor deficit intermediate phenotype (9). As a result, M_1_ receptors are now considered an important focus of the neurobiology and treatment of SZ.

Until recently, investigations of muscarinic function in SZ have been mostly limited to postmortem studies, which have consistently demonstrated reductions in cortical muscarinic M_1_ receptors, most notably in the dorsolateral prefrontal cortex. A subgroup (~25%) showed ~70% to 75% lower binding of the muscarinic antagonist [^3^H]pirenzepine (>80% of binding attributable to M_1_ over M_4_) compared with control participants (10). Further work linked these reductions to altered M_1_ messenger RNA, promotor methylation, and microRNA regulation (11). These postmortem findings raise the intriguing possibility that M_1_ hypofunction may contribute to the neurobiology of a muscarinic receptor–deficit SZ subtype.

In vivo binding studies with single-photon emission computed tomography (SPECT) radioligands (nonselective M_1_/M_4_ antagonists: ^123^I-IQNB, ^123^I-IDEX) have shown lower binding in patients with SZ [as reviewed in (12)]. M_1_ receptors can now be imaged in vivo at higher resolution with positron emission tomography (PET) and the radiotracer ^11^C-LSN3172176, an M_1_ agonist that reflects the high-affinity state of receptors. ^11^C-LSN3172176 has excellent kinetic properties, as shown in healthy control (HC) participants: relatively high M_1_ selectivity, high brain uptake (peak standardized uptake values of 4 to 9), high specific binding, and high test-retest reliability (<10% variability in all regions), with the cerebellum identified as a suitable reference region (13). In the current study, to test the hypothesis of M_1_ receptor deficits in SZ, in vivo brain muscarinic M_1_ receptor availability was measured in SZ for the first time using ^11^C-LSN3172176.

## METHODS AND MATERIALS

### Regulatory

The study was performed under protocols approved by the Yale Human Investigation Committee, the Yale University Radiation Safety Committee, the Yale University Radioactive Drug Research Committee, and the Yale MRI Safety Committee.

### Participants

Individuals diagnosed with DSM-5 SZ and age- and sex-matched HCs were recruited. All participants had a comprehensive screening assessment that included the Structured Clinical Interview for DSM-5 Axis I disorders, Clinician Version, to confirm diagnosis, medical history, physical examination, routine blood tests, and electrocardiogram and urine toxicology (see Supplemental Methods for detailed inclusion and exclusion criteria). Patients currently on potent or M_1_-selective anticholinergic medications were excluded.

HCs consisted of new participants and historical control participants from the Yale database of studies with ^11^C-LSN3172176 (*n* = 3). Most participants (*n* = 11 from both groups) were scanned during the same period (June 2021–December 2022).

### Magnetic Resonance Imaging

Structural magnetic resonance (MR) imaging was performed on a Siemens 3T Trio system (Siemens Medical Solutions) for purposes of excluding individuals with anatomical abnormalities and anatomically coregistering with PET scans. The dimension and voxel size of MR images were 256 × 256 × 176 voxels and 0.98 × 0.98 × 1.0 mm^3^, respectively.

### Radiochemistry, PET Acquisition, and Arterial Blood Sampling

^11^C-LSN3172176 was synthesized at the Yale PET Center as previously reported (13). All ^11^C-LSN3172176 PET measurements were conducted on the High-Resolution Research Tomograph (Siemens Medical Solutions), which acquires 207 slices (1.2-mm slice separation) with a reconstructed image resolution (full width at half maximum) of ~3 mm. Before every ^11^C-LSN3172176 injection, a 6-minute transmission scan was performed for attenuation correction. PET data were acquired in list mode for 120 minutes after the start of the ^11^C-LSN3172176 administration.

The dynamic emission data were reconstructed into 33 frames (6 × 0.5, 3 × 1, 2 × 2, and 22 × 5 minutes) with corrections for attenuation, normalization, scatter, randoms, and dead time using the motion-compensation OSEM list-mode algorithm for resolution-recovery reconstruction (MOLAR) (14). Event-by-event motion correction (15) was included in the reconstruction on the basis of motion detection with a Polaris Vicra optical tracking system (NDI) using markers mounted on a swim cap worn by the participant.

In a subset of participants (*n* = 25), discrete blood samples were manually drawn and centrifuged to obtain plasma, and then whole blood and plasma were counted with a calibrated well counter. Metabolites were analyzed using the column-switching high-performance liquid chromatography method (16) to determine the parent fraction (see Supplemental Methods). An ultrafiltration-based method was used for measuring the plasma-free fraction (*f*_P_), as previously described (17).

### Image Registration and Definition of Regions of Interest

For each participant, the reconstructed dynamic PET data were coregistered to an early summed PET image (0–10 minutes after ^11^C-LSN3172176 injection) using a 6-parameter mutual information algorithm (FLIRT of FSL 3.2) to eliminate any residual motion. The summed PET image was coregistered to the participant’s individual T1-weighted 3T MR image (6-parameter rigid registration) and subsequently coregistered to the Automated Anatomical Labeling (AAL) template in Montreal Neurological Institute space using nonlinear transformation (BioImage Suite 2.5) (18). Regions of interest (ROIs) were taken from the AAL template. The following ROIs were included in the primary analysis: frontal, temporal, parietal, and occipital cortices; caudate; putamen; pallidum; thalamus; cerebellum; hippocampus; and amygdala. ROI percent gray matter (%GM) fractions were calculated from CAT12 tissue segmentations and evaluated to account for potential GM atrophy related to SZ pathology and/or age.

### Quantitative Analysis of Binding

Time-activity curves were generated with the AAL atlas for the main cortical ROIs and analyzed using kinetic modeling. Specifically, distribution volume (*V*_T_) estimates (19) were generated using the 1-tissue compartment model (1T) (13) for the subset of participants with arterial sampling. *V*_T_ is the ratio of total ligand uptake in tissue relative to the total concentration of ligand in plasma at equilibrium, including both specific and nondisplaceable components. On the other hand, the distribution volume ratio (*DVR*) is the ratio of the total radioligand (specific plus nonspecific) to that of nondisplaceable radioligand in tissue (19). *DVR*_CS_ was calculated using the centrum semiovale (CS) as the pseudoreference region using the simplified reference tissue model with parameter coupling (20,21). A small CS region (~2 mL) was used to reduce potential spill-in and partial volume effects. The ROI size was chosen based on what was reported by Rossano *et al.* (22) for ^11^C-UCB-J, without additional optimization for this tracer. The reasoning for using the CS rather than the cerebellum [as in (13)] as the reference region is outlined in Results. Whole-brain average *DVR*_CS_ and *V*_T_ values (mean of the chosen ROIs) were also calculated. All kinetic modeling was performed with in-house developed programs written with IDL 8.0 (ITT Visual Information Solutions) (see Supplemental Methods). Data from GM-segmented ROIs (CAT12 segmentations) and data corrected for partial volume effects using the Müller-Gartner partial volume correction algorithm (23) were also similarly quantified.

### Assessments

Participants were assessed by chart review and a complete psychiatric and physical examination. The severity of SZ symptoms was assessed using the Positive and Negative Syndrome Scale (PANSS) (24). Verbal learning and memory were assessed with the Rey Auditory Verbal Learning Test (RAVLT), while other cognitive functions were assessed with the CogState cognitive test battery (25,26) which includes the detection test, identification test, one-back test, international shopping list, Groton Maze Learning Test, and social-emotional cognition test (see Supplemental Methods). Current antipsychotic exposure was calculated using standardization to chlorpromazine equivalents (27). The Fagerström Test for Nicotine Dependence (FTND) (28) was used to assess the severity of nicotine dependence, and recent alcohol/substance use was assessed using the Timeline Follow-Back Scale (29).

### Statistical Analysis

Initially, data were examined descriptively using means, standard deviations, and graphs. Based on examination of normal probability and residual plots and Kolmogorov-Smirnov test statistics, each outcome appeared normal for parametric analyses. Linear mixed-effects (LME) models were used to model the independent and joint effects of diagnosis (SZ vs. HC) and ROI (within-subjects factor) on *DVR*_CS_ and *V*_T_ values, accounting for the effect of covariates if necessary (ROI-wise %GM included as an additive linear covariate, as well as smoking or cannabis use status, antipsychotic exposure). ROI-specific contrasts were derived from the interaction model (Wald tests), and *p* values were corrected for multiple comparisons across ROIs using the Benjamini-Hochberg false discovery rate (FDR) procedure. See Supplemental Methods for more details. Effect sizes were calculated as Cohen’s *d*. Individual patient differences (%diff_i_) in whole-brain M_1_ availability (whole-brain mean of *DVR*_CS_ and *V*_T_ across ROIs) against the HC mean values were calculated.

The relationship between M_1_ availability in brain regions and symptomatology was explored in the SZ group. Associations between whole-brain and regional *DVR*_CS_ values and measures of illness severity (age of onset of SZ, illness duration), antipsychotic exposure, tobacco and cannabis use, psychopathology (PANSS scores), and performance on cognitive measures were evaluated using *t* tests and Pearson’s or partial correlations (adjusting for %GM fraction). Given their exploratory nature, these analyses were not corrected for multiple comparisons.

## RESULTS

An equal number (*n* = 16) of patients with SZ and age- and sex-matched HCs were scanned (Table 1). There were no statistically significant differences between groups on age, sex, body mass index, injected dose of ^11^C-LSN3172176, mass dose (ng/kg and μg), molar activity at the time of injection, or *f*_P_ of ^11^C-LSN3172176 (Table 1). In the SZ group, the mean ± SD age of onset of illness was 24.44 ± 5.37 years, and the mean duration of illness was 13.69 ± 13.31 years (Table 2). Most patients were taking second-generation antipsychotics: brexpiprazole (*n* = 1), risperidone (*n* = 2), lurasidone (*n* = 2), or aripiprazole (*n* = 5). Only 2 patients were taking first-generation antipsychotics (haloperidol [*n* = 2], perphenazine [*n* = 1]), and 3 patients were antipsychotic free. The affinity (*K*_i_, nM) of each antipsychotic for M_1_ receptors is reported in Table 2. Arterial blood was sampled in most participants (SZ, *n* = 11; HC, *n* = 14) (Figure S1). Five patients with SZ were smokers, but only 2 met criteria for nicotine dependence (FTND score >0), 1 with a score of 5 (moderate dependence) and 1 with a score of 8 (high dependence); none of the HCs were smokers (SZ vs. HC: Fisher’s exact test, *p* = .04). Four patients with SZ had recently used cannabis (previous 30 days), but only 2 of them used every day; none of the HCs were current cannabis users (SZ vs. HC: Fisher’s exact test, *p* = .10) (Table 1).

There was no significant group difference in ^11^C-LSN3172176 distribution volume (*V*_T_) (mL/cm^3^) in the CS white matter (mean ± SD in SZ: 5.76 ± 1.13, HC: 5.54 ± 0.66; *t* test, *p* = .55), supporting its use as a pseudoreference region. In contrast, cerebellum *V*_T_ was lower in the SZ group than in the HC group (mean ± SD in SZ: 3.85 ± 0.74, HC: 4.47 ± 0.66; *t* test, *p* = .04), as was cerebellum %GM (mean ± SD in SZ: 62.51% ± 4.65%, HC: 68.79% ± 4.08%; *t* test, *p* = .003) (see Supplemental Results; Figures S2 and S3). While there was no significant group difference in %GM (main effect of diagnosis: *F*_1,166_ = 1.05, *p* = .307), there was a significant diagnosis × ROI interaction (*F*_10, 320_ = 3.46, *p* < .001). The ROI-wise %GM values and LME contrasts are reported in Table S1.

### Distribution Volumes in Participants With SZ Versus HCs

**Figure 1.**
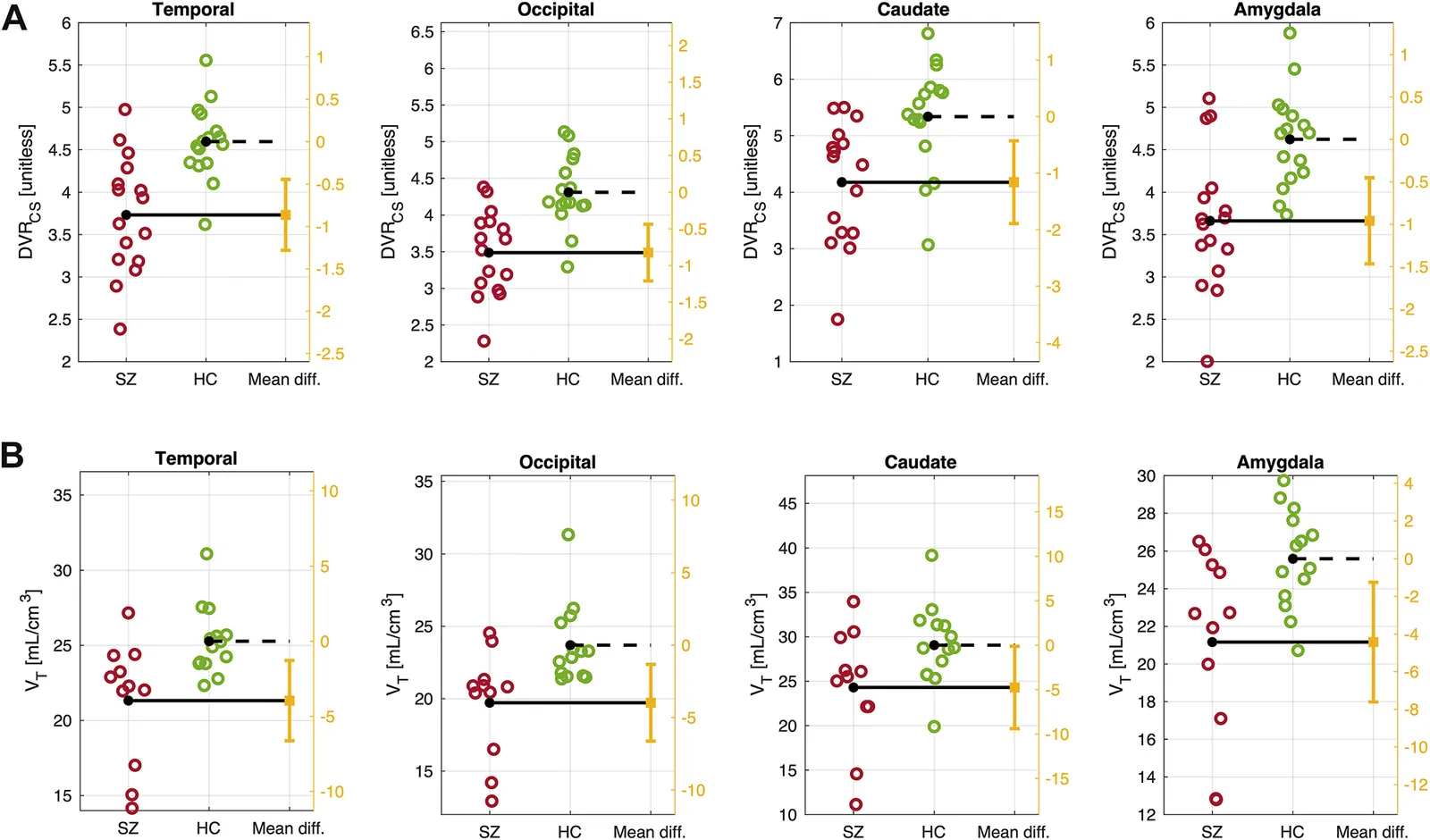
Comparison of M_1_ receptor availability [^11^C-LSN3172176 *DVR*_CS_ **(A)** and *V*_T_ **(B)**] in patients with SZ and HC participants across representative regions of interest. M_1_ receptor density as measured by ^11^C-LSN3172176 availability (*DVR*_CS_, *V*_T_ [mL/cm^3^]) (y-axis, left). Patients with SZ [**(A)** *n* = 16, **(B)** *n* = 11] shown in red, HC participants [**(A)** *n* = 16, **(B)**, *n* = 14] shown in green. The mean of the HC participants (dashed black line) corresponds to the 0 mean difference (y-axis, right, yellow). The mean difference value (corresponding to the mean of the SZ sample, continuous black line) and CIs (vertical error bar) are displayed. *DVR*_CS_, distribution volume ratio relative to the centrum semiovale; HC, healthy control; SZ, schizophrenia.

The main PET outcome parameter was *DVR*_CS_ complemented by evaluation of 1T total distribution volume (*V*_T_). Patients with SZ had significantly lower ^11^C-LSN3172176 binding (*DVR*_CS_) than HCs (main effect of diagnosis: *F*_1,81_ = 23.4, *p* < .001; main effect of ROI: *F*_10,320_ = 330.12, *p* < .001; diagnosis × ROI interaction: *F*_10,320_ = 9.04, *p* < .001). This was similar for *V*_T_ (main effect of diagnosis: *F*_1,65_ = 11.02, *p* = .001; main effect of ROI: *F*_10,250_ = 253.71, *p* < .001; diagnosis × ROI interaction: *F*_10,250_ = 3.83, *p* < .001). %GM showed a strong effect on the outcome measures (*DVR*_CS_: *F*_1,340_ = 83.74, *p* < .001; *V*_T_: *F*_1,263_ = 70.81, *p* < .001). Model-derived contrasts revealed widespread reductions in the SZ group relative to the HC group across multiple ROIs, even after controlling for %GM (Figure 1; Tables 3 and 4), with the exception of the cerebellum, thalamus, and pallidum (all regions with low specific binding). Notably, 7 of 16 (44%) patients with SZ had whole-brain *DVR*_CS_ %diff_i_ larger than 20%, and 3 of the 11 (27%) patients had whole-brain *V*_T_ %diff_i_ larger than 20%. ROI-wise contrasts for *DVR*_CS_ and *V*_T_ without accounting for %GM differences are reported in Table S2. ROI-wise contrasts for *V*_T_ normalized by *f*_P_ (*V*_T_/*f*_P_) are reported in Table S3.

*DVR*_CS_ was negatively correlated with age in patients with SZ (whole brain: *r* = −0.61, *p* = .01) and in the whole group (*r* = −0.47, *p* = .007) but not in HCs (*r* = −0.31, *p* = .24). However, including age as an additional covariate in the LME models did not affect results for either *DVR*_CS_ or *V*_T_ (Table S4). *DVR*_CS_ and *V*_T_ results obtained with %GM as a covariate were highly consistent with GM-masked and partial volume–corrected results (Supplemental Results; Tables S5 and S6).

Including smoking status (Table 1) as an additional covariate to %GM did not impact the significance of the main effects of diagnosis (*DVR*_CS_: *F*_1,51_ = 17.18, *p* < .001; *V*_T_: *F*_1,51_ = 5.87, *p* = .018) or the diagnosis × ROI interaction (*DVR*_CS_: *F*_10,320_ = 12.43, *p* < .001; *V*_T_: *F*_10,250_ = 6.25, *p* < .001), although it affected the ROI-wise contrast significance for *V*_T_ (Table S7). Moreover, group differences persisted after excluding nicotine-dependent smokers (Table S8). Similarly, covarying for cannabis use status (current users, past 30 days) did not impact the significance of the main effects of diagnosis (*DVR*_CS_: *F*_1,51_ = 21.50, *p* < .001; *V*_T_: *F*_1,55_ = 6.63, *p* = .013) or the diagnosis × ROI interaction (*DVR*_CS_: *F*_10,320_ = 12.46, *p* < .001; *V*_T_: *F*_10, 250_ = 6.29, *p* < .001), but it affected the ROI-wise contrast significance for *V*_T_ (Table S9).

### Exploratory Analyses Relating M_1_ Availability to Symptomatology and Disease Characteristics in Patients With SZ

**Figure 2.**
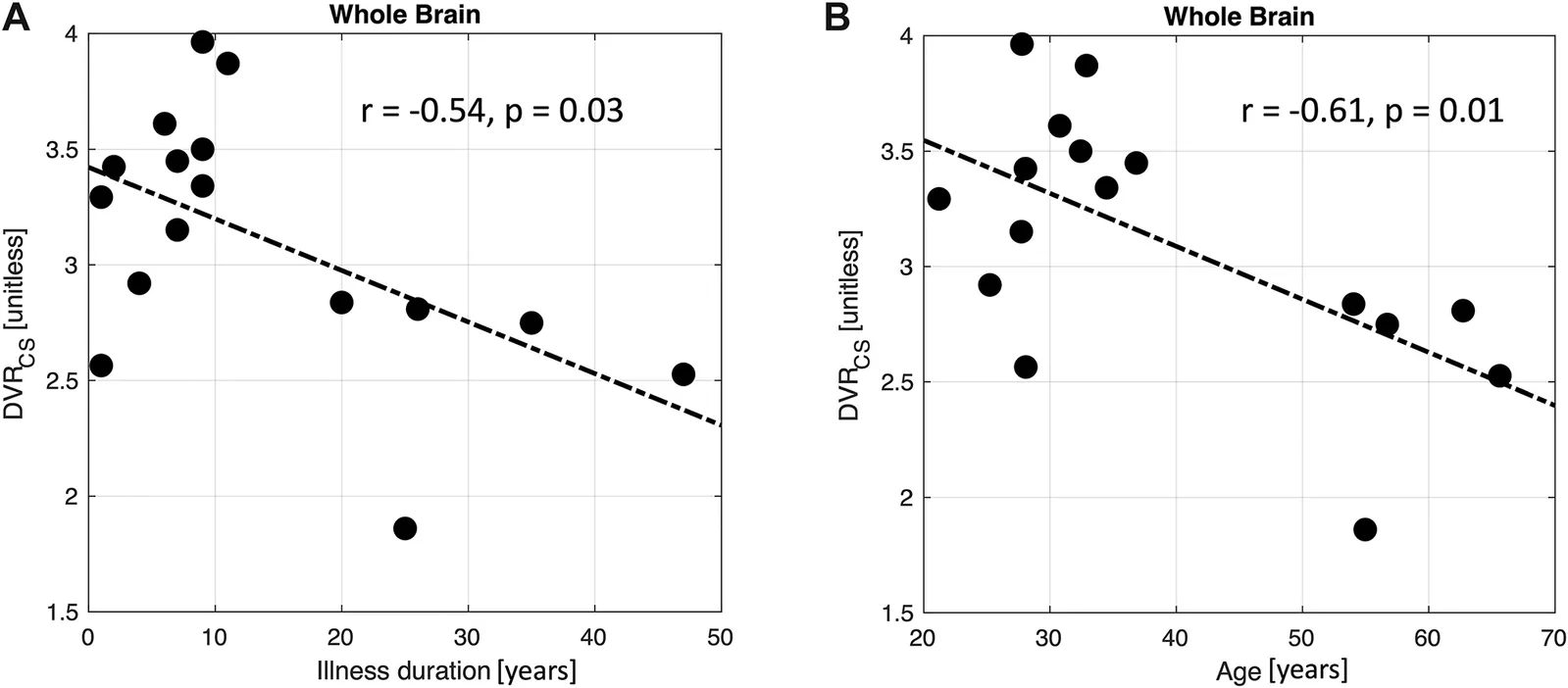
Relationship between M_1_ receptor availability (whole-brain *DVR*_CS_) and illness duration **(A)** and age **(B)** in schizophrenia. *DVR*_CS_, distribution volume ratio relative to the centrum semiovale.

The whole-brain mean *DVR*_CS_ and the *DVR*_CS_ of multiple individual ROIs were negatively correlated with illness duration (whole brain: *r* = −0.54, *p* = .03; frontal: *r* = −0.61, *p* = .01; temporal: *r* = −0.59, *p* = .02; parietal: *r* = −0.61, *p* = .01; occipital: *r* = −0.54, *p* = .03; caudate: *r* = −0.65, *p* = .01; hippocampus: *r* = −0.53, *p* = .03) (Figure 2). Some individual ROI correlations survived adjustment for %GM (frontal: *r* = −0.55, *p* = .04; temporal: *r* = −0.57, *p* = .03; parietal: *r* = −0.53, *p* = .04; occipital: *r* = −0.54, *p* = .05).

There were no significant effects of current antipsychotic dose on whole-brain *DVR*_CS_ or *V*_T_/*f*_P_, but there was a negative correlation with *V*_T_ (*r* = −0.73, *p* = .02) (Figure S4). Including current antipsychotic exposure as an additional covariate to %GM did not eliminate the significance of the main effects of diagnosis for *DVR*_CS_ (*F*_1,61_ = 6.96, *p* = .011) or the diagnosis × ROI interaction (*F*_10,320_ = 12.47, *p* < .001), although it affected the ROI-wise contrast significance (Table S10) and made the main effect of diagnosis not significant for *V*_T_ (*F*_1,50_ = 6.25, *p* = .64).

SZ patients who were smokers (*n* = 5) and nonsmokers did not differ significantly in whole-brain *V*_T_ (*t* test, *p* = .18, 16% mean difference from nonsmokers) or *DVR*_CS_ (*t* test, *p* = .08, 15% difference), although there was a trend. The only 2 patients with FTND total scores >0 tended to have lower *DVR*_CS_. Likewise, patients with SZ recently exposed to cannabis (*n* = 4) did not differ significantly in whole-brain *V*_T_ (*t* test, *p* = .36, 13% mean difference from nonusers) or *DVR*_CS_ (*t* test, *p* = .88, 2% mean difference). No significant correlation with lifetime exposure to cannabis or other substances was detected.

**Figure 3.**
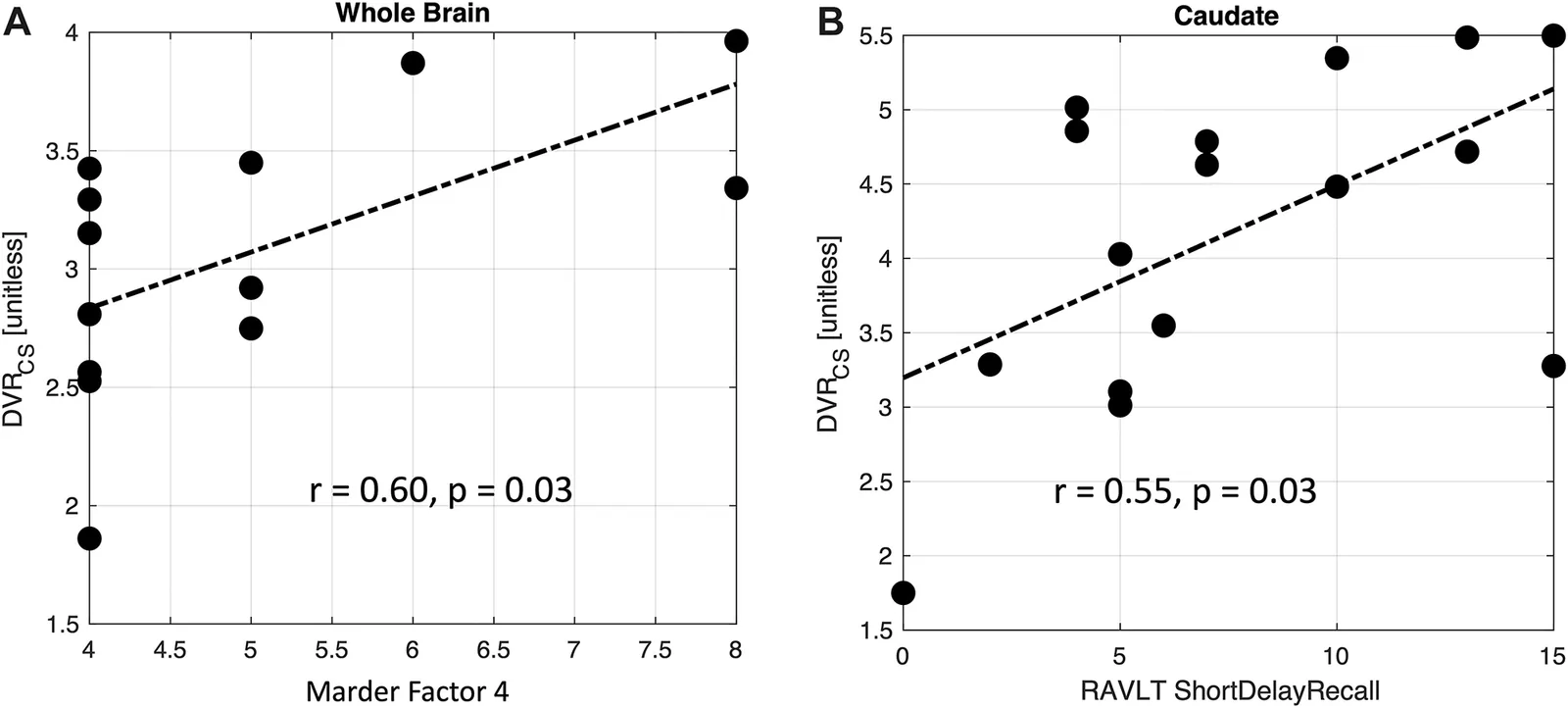
Relationship between M_1_ receptor availability and **(A)** symptom severity (whole-brain *DVR*_CS_ vs. Positive and Negative Syndrome Scale, Marder Factor 4, uncontrolled hostility/excitement) and **(B)** memory (caudate *DVR*_CS_ vs. RAVLT short-delay recall) in schizophrenia. *DVR*_CS_, distribution volume ratio relative to the centrum semiovale; RAVLT, Rey Auditory Verbal Learning Test.

**Figure 4.**
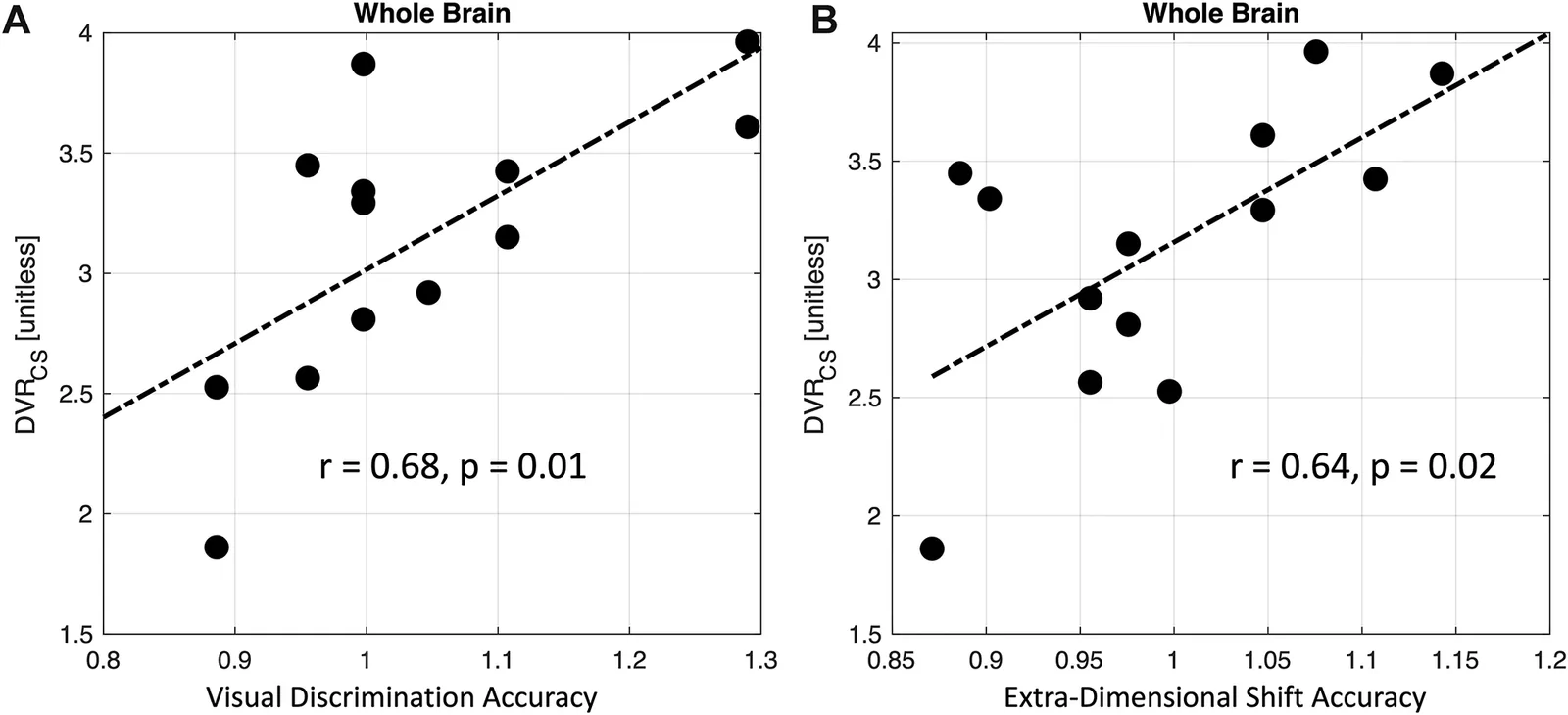
Relationship between M_1_ receptor availability (whole-brain *DVR*_CS_) and CogState items in schizophrenia: visual discrimination accuracy **(A)** and extradimensional shift accuracy **(B)**. *DVR*_CS_, distribution volume ratio relative to the centrum semiovale.

The whole-brain mean *DVR*_CS_ and the *DVR*_CS_ of multiple individual ROIs were positively correlated with the PANSS Marder Factor 4 uncontrolled hostility/excitement dimension (whole brain: *r* = 0.60, *p* = .03) (Figure 3A). Notably, the correlations were strengthened by accounting for %GM. Caudate *DVR*_CS_ was positively correlated with RAVLT short-delay recall (*r* = 0.55, *p* = .03) (Figure 3B). Among CogState primary outcomes, whole-brain *DVR*_CS_ was correlated with performance in visual discrimination (accuracy: *r* = 0.68, *p* = .01) (Figure 4A), extradimensional shift (accuracy: *r* = 0.61, *p* = .02) (Figure 4B), and Groton Maze learning (duration: *r* = −0.68, *p* = .001) and recall (duration: *r* = −0.61, *p* = .03).

## DISCUSSION

This study provides the first in vivo PET evidence of lower muscarinic M_1_ receptor availability in SZ. The overlap between the regional pattern of deficits observed here and postmortem evidence further support the plausibility of a muscarinic receptor deficit (9) and M_1_ as a treatment target for SZ. The findings are particularly timely given the recent FDA approval of xanomeline-trospium, the first non–dopamine D_2_-based medication for SZ that has a muscarinic M_1_/M_4_ mechanism of action. Differences between patients with SZ and HCs were large (Cohen’s *d* > 1) and across multiple brain regions. Furthermore, these differences were observed using relative and absolute kinetic outcomes (*DVR*_CS_ and *V*_T_), supporting robustness across modeling approaches and strengthening confidence in the validity of the findings.

Several design and analytic features reduce the likelihood that the observed differences are attributable to confounding. The groups were matched for age and sex (and including age as an additional covariate did not affect results). Results obtained with regionwise GM fraction included as a covariate to mitigate the influence of differential atrophy on PET signal were fully consistent with GM-masked and partial volume–corrected results (30). Medication effects are also unlikely to fully explain the findings. Participants taking potent or M_1_-selective anticholinergic agents (e.g., benztropine, trihexyphenidyl) and antipsychotics with high M_1_ affinity (e.g., olanzapine or clozapine) were excluded. M_1_ binding was not robustly associated with antipsychotic dose or antipsychotic class, at least when estimated as *DVR*_CS_ and *V*_T_/*f*_P_, but there was a negative correlation between antipsychotic exposure and *V*_T_. Another potential confounder is tobacco smoking, given its impact on ACh receptors in SZ [especially nicotinic (31,32)]. Only a minority of patients (*n* = 2) met criteria for nicotine dependence, and group differences persisted after accounting for smoking status and excluding smokers with nicotine dependence; these findings argue against smoking as the primary driver of group differences in M_1_ binding. Cannabis use, which has been associated with M_1_ deficits in preclinical studies (33), is also unlikely to be the primary driver of M_1_ group differences, because it was fairly limited in most participants, and no association with lifetime or recent exposure was detected. Nonetheless, further investigation of the effects of antipsychotic exposure and smoking tobacco and cannabis on ^11^C-LSN3172176 binding with larger cohorts is warranted.

A subset of patients (~30%–45%) showed large (>20%) reductions in M_1_ availability relative to matched control participants, findings that may be consistent with the postmortem evidence that a subgroup (~25%) showed greatly lower binding of an M_1_ antagonist compared with the control group (9,10). However, a recent meta-analysis yielded less definitive support for the presence of such a distinct subgroup (8). Larger PET cohorts will be necessary to definitively establish whether M_1_ deficits are best characterized as a categorical subtype, a continuous dimension, or a combination of both.

Exploratory analyses revealed correlations between M_1_ availability and several clinical features in SZ. M_1_ receptor availability correlated with performance across multiple cognitive domains, including verbal memory (RAVLT), visual discrimination, visuospatial memory (Groton Maze learning and recall), and executive functioning (extradimensional shift). These findings are consistent with prior SPECT studies demonstrating that reduced M_1_/M_4_ radioligand binding in the frontal cortex and hippocampus in medication-free individuals with psychosis is associated with impairments in verbal learning and memory (34). They are also consistent with converging preclinical and clinical evidence supporting procognitive effects of M_1_ agonists, including xanomeline-trospium (35–37). Furthermore, M_1_ availability was also positively associated with the PANSS hostility/excitement dimension, an observation that is particularly intriguing given clinical trial evidence that xanomeline-trospium reduces agitation in acutely ill patients with SZ (38). Although these analyses are exploratory, they raise the possibility that M_1_ receptor availability may index clinically meaningful variation in both cognitive and behavioral symptom domains and could have relevance for predicting response to emerging muscarinic-based treatments.

These findings warrant further investigation of M_1_ availability in SZ. First, further characterization of the relationship between M_1_ availability and disease characteristics including positive, negative, and cognitive symptoms will be important, given initial evidence that xanomeline-trospium improves cognitive impairment in SZ patients with cognitive deficits (36) and reduces agitation in acutely ill patients with SZ (38). Second, longitudinal studies are needed to determine whether reduced M_1_ availability is present early in illness, changes with disease course, and whether it is better conceptualized as a trait marker or a state-dependent feature that evolves with chronicity, particularly given the observed association with illness duration in exploratory analyses. Third, the role of dopamine D_2_ antipsychotic medication status should be clarified through studies that include carefully characterized samples on and off antipsychotic treatment, while accounting for smoking and substance exposure. Finally, the translational relevance of M_1_ PET is highlighted by the approval of xanomeline-trospium and the development of other muscarinic agonist drugs currently in development, including M_1_ and M_4_ positive allosteric modulators [e.g., ANAVEX3–71 (39), emraclidine (NCT07145918)] and dual M_1_/M_4_ agonists [e.g., ML-007 (40)]. M_1_ availability could plausibly serve as a biomarker to predict treatment response, tolerability, or optimal patient selection. Critically, this remains an empirical question, as it remains unclear whether patients with the largest M_1_ deficits will derive the greatest benefit from agonist strategies or whether preserved M_1_ receptor availability is necessary for robust pharmacological response. This underscores the value of prospective biomarker-stratified studies (41).

Several limitations warrant consideration. The relatively small sample size limits power for subgroup detection and symptom correlations and restricts evaluation of sex effects. The choice of reference region requires particular caution (Supplemental Results); although the cerebellum has been used as a reference region in prior work (13), the presence of group differences in cerebellar *V*_T_ and GM fraction in the current sample rendered it unsuitable, motivating the use of the CS as a pseudoreference region and estimation of *DVR* rather than nondisplaceable binding potential (*BP*_ND_). This is consistent with ample evidence of GM volume changes in SZ (42). Nonetheless, a disease-related effect on nondisplaceable binding (*V*_ND_), as reflected by cerebellar *V*_T_, cannot be excluded. Future studies should include additional validation of the cerebellum and CS in older and clinical samples with scopolamine blocking or leverage emerging noninvasive methods (e.g., image-derived input functions) to enable *V*_T_ estimation without arterial sampling and reduce dependence on reference-region assumptions (43,44). Finally, although ^11^C-LSN3172176 has its highest affinity for M_1_ receptors (*K*_i_ = 8.9 nM) (13), it also shows measurable affinity for M_4_ (*K*_i_ = 41.4 nM) and M_2_ (*K*_i_ = 63.8 nM), raising the possibility of some non-M_1_ contribution to the PET signal in the striatum, where M_4_ receptors are relatively abundant (45,46). Available postmortem binding data in HCs suggest an approximate 2:1 ratio of striatal M_1_ to combined M_2_/M_4_ sites, and simple affinity-weighted estimates suggest that non-M_1_ binding may account for a modest fraction of the striatal signal (up to 10%–15%). Accordingly, striatal ^11^C-LSN3172176 binding should be interpreted as predominantly M_1_ weighted rather than purely M_1_ specific, pending subtype-selective (M_4_/M_2_) blocking studies with both HCs and patients with SZ (who could have different non-M_1_ contribution). Also, we previously showed that occupancy values generated with scopolamine partial blocking were lower in the striatum than in nonstriatal regions (13), which may be attributable to regional differences in endogenous ACh concentrations (47). Future work should attempt to characterize how regional variations in ACh concentration differ between patients with SZ and HCs using the same blocking paradigm.

## Conclusions

This study provides the first in vivo PET evidence of reductions in muscarinic M_1_ receptor availability in SZ, supporting and extending decades of postmortem findings. Using the M_1_-preferring PET radiotracer ^11^C-LSN3172176, we demonstrated robust group differences across multiple cortical and subcortical regions, with large effect sizes and a substantial subset of patients exhibiting marked M_1_ deficits. These findings are timely, given the recent approval of the M_1_/M_4_ agonist xanomeline-trospium. Exploratory associations with illness duration and cognitive symptoms suggest clinical relevance and warrant larger and longitudinal studies to clarify state versus trait effects and treatment relationships. The effects of sex, smoking, and antipsychotic exposure will require further characterization. Together, these results establish muscarinic M_1_ receptor availability as a promising in vivo biomarker for SZ pathophysiology and lay the groundwork for precision approaches to patient stratification and treatment selection in the emerging era of nondopaminergic therapeutics.

## Supplementary Material

Supplementary Material

Supplementary material cited in this article is available online at https://doi.org/10.1016/j.biopsych.2026.06.002.

## Figures and Tables

**Table 1.** Demographics of schizophrenia (SZ) patients and healthy controls (HC)

	Diagnosis	*N*	Mean	SD	*p* value
Age	HC	16	36.78	10.01	0.66
	SZ	16	38.73	14.69	
Sex (F/M)	HC	2/14			1.00
	SZ	2/14			
BMI	HC	16	25.63	2.52	0.06
	SZ	16	29.12	6.56	
Tobacco smokers (yes/no)	HC	0/16			0.04*
	SZ	5/11			
Cannabis recent users (yes/no)	HC	0/16			0.10
	SZ	4/12			
Injected dose (MBq)	HC	16	549.01	151.11	0.90
	SZ	16	541.73	185.41	
Mass dose (μg)	HC	16	1.04	0.88	0.67
	SZ	16	1.16	0.68	
Mass dose (ng/kg)	HC	16	12.80	9.64	0.99
	SZ	16	12.82	6.58	
Molar activity at time of injection (MBq/nmol)	HC	16	279.07	124.52	0.14
	SZ	16	217.24	104.68	
Plasma free fraction (*f*_P_)	HC	16	0.28	0.02	0.34
	SZ	15	0.28	0.03	

**Table 2.** Clinical characteristics of schizophrenia (SZ) patients

Characteristics	*N*	Mean	SD
Age at onset of illness	16	24.44	5.37
Duration of illness	16	13.69	13.31
PANSS total	15	64.20	18.55
PANSS positive	15	17.53	6.14
PANSS negative	15	15.60	6.74
PANSS general	15	31.07	8.00
Antipsychotic exposure (CPZ equivalent dose x day (mg/day))	12	337.50	143.35
Haloperidol (M1 *K*_i_ > 10,000)	2		
Perphenazine (M1 *K*_i_: 2,000)	1		
Brexpiprazole (M1 *K*_i_ > 1,000)	1		
Risperidone (M1 *K*_i_ > 10,000)	2		
Lurasidone (M1 *K*_i_ > 1,000)	2		
Aripiprazole (M1 *K*_i_: 6,780)	5		
Antipsychotic Free	3		

**Table 3.** Group Differences in M1 receptor availability ^11^C-LSN3172176 *DVR*_CS_ in schizophrenia (SZ) patients vs. healthy controls (HC) adjusted for differences in percent gray matter (GM))

		*N*	Mean	SD	*p* value (FDR)	% diff.	Cohen d
Frontal	HC	16	4.08	0.35	0.004*	−13	1.21
	SZ	16	3.45	0.62			
Temporal	HC	16	4.60	0.44	<0.001*	−15	1.45
	SZ	16	3.73	0.69			
Parietal	HC	16	4.06	0.43	0.003*	−14	1.15
	SZ	16	3.41	0.65			
Occipital	HC	16	4.31	0.49	0.001*	−16	1.50
	SZ	16	3.49	0.58			
Caudate	HC	16	5.34	0.95	<0.001*	−19	1.12
	SZ	16	4.18	1.08			
Putamen	HC	16	6.51	0.56	<0.001*	−19	1.32
	SZ	16	5.39	1.03			
Pallidum	HC	16	2.42	0.33	0.309	−8	0.24
	SZ	16	2.35	0.29			
Thalamus	HC	16	1.60	0.13	0.829	−3	1.17
	SZ	16	1.40	0.19			
Hippocampus	HC	16	3.04	0.38	0.028*	−13	1.29
	SZ	16	2.47	0.48			
Amygdala	HC	16	4.62	0.57	<0.001*	−19	1.33
	SZ	16	3.66	0.81			
Cerebellum	HC	16	0.85	0.10	0.494	16	0.97
	SZ	16	0.75	0.10			

**Table 4.** Group Differences in M1 receptor availability ^11^C-LSN3172176 *V*_T_ in schizophrenia (SZ) patients vs. healthy controls (HC) (adjusted for differences in percent gray matter (GM))

		*N*	Mean	SD	*p* value (FDR)	% diff.	Cohen d
Frontal	HC	14	22.17	1.83	0.117	−10	0.86
	SZ	11	19.68	3.71			
Temporal	HC	14	25.28	2.26	0.036*	−12	1.19
	SZ	11	21.32	4.12			
Parietal	HC	14	22.27	2.23	0.087	−11	1.00
	SZ	11	19.37	3.40			
Occipital	HC	14	23.70	2.73	0.031*	−13	1.21
	SZ	11	19.72	3.69			
Caudate	HC	14	29.06	4.48	0.002*	−15	0.83
	SZ	11	24.30	6.71			
Putamen	HC	14	36.30	3.88	<0.001*	−17	0.85
	SZ	11	31.03	7.93			
Pallidum	HC	14	13.30	2.19	0.439	−9	0.04
	SZ	11	13.18	3.43			
Thalamus	HC	14	8.52	0.87	0.879	−2	1.22
	SZ	11	7.22	1.20			
Hippocampus	HC	14	16.34	1.53	0.208	−11	1.19
	SZ	11	13.77	2.64			
Amygdala	HC	14	25.59	2.61	0.005*	−16	1.12
	SZ	11	21.16	4.96			
Cerebellum	HC	14	4.47	0.66	0.463	21	0.87
	SZ	11	3.85	0.74			
