# Supplementary material for: Lower Muscarinic M1 Receptor Availability in Schizophrenia: In Vivo Positron Emission Tomography Evidence
Source: Biol Psychiatry. Author manuscript; Available in PMC 2026 Aug 27. (PMC13507943; doi:10.1016/j.biopsych.2026.06.002)
Supplement: Supplementary file 1 [file NIHMS2193328-supplement-Supplementary_Material.pdf]

SUPPLEMENTARY INFORMATION

Lower Muscarinic M1 Receptor Availability in Schizophrenia: In Vivo Positron Emission Tomography Evidence

Volpi *et al.*

Supplementary Material:

Supplementary Methods:

Exclusion criteria in schizophrenia (SZ) patients and healthy controls (HC)

SZ subjects were excluded for the following reasons:

1. Regular exposure to drugs of abuse (except nicotine and caffeine) within the past 3 months (based on history) or positive urine drug screen,
2. Lifetime substance use disorder (except for nicotine and caffeine),
3. Weekly alcohol consumption exceeding National Institute on Alcohol Abuse and Alcoholism (NIAAA) guidelines (4 or more drinks on any single day and/or 21 or more drinks per week for males),
4. Current exposure to anticholinergic medications (whenever possible); for SZ: use of antipsychotic medications that have limited affinity for the muscarinic M1 receptor (such as Risperidone, Paliperidone, Iloperidone, Asenapine, Amisulpride, Aripiprazole, Brexipiprazole, Cariprazine, Haloperidol, Lurasidone, Ziprasidone),
5. History of significant head injury resulting in unconsciousness,
6. Unstable medical condition,
7. Significant prior exposure to radiation,
8. Metal in the body which would exclude MRI scan,
9. Claustrophobia.

Healthy control (HC) individuals were excluded for a current and/or lifetime diagnosis of a psychiatric disorder, current or past serious medical or neurological illness (including a history of head injury with loss of consciousness), a history of substance use disorder, illicit drug use in the past 3 months, metal in their body (which would exclude MRI scanning) and claustrophobia and significant prior exposure to radiation.

Drug and alcohol use was assessed by careful review of the medical record, contact with the mental health clinician, SCID for DSM-5 diagnosis (including substance use disorders), and urine toxicological testing using a 9-panel urine drug screen (that detects amphetamines, benzodiazepines, barbiturates, cannabinoids, cocaine, methadone, opioids, amphetamines, PCP) on 2 occasions (screening and PET scan day), and the Fagerstrom Test for Nicotine Dependence (FTND). A few subjects were regular tobacco smokers. However, none of the subjects met DSM-5 criteria for current substance-use disorder or had urine toxicology tests positive for cannabis or other street drugs or were regular users of street drugs.

Arterial blood Sampling and kinetic analysis:

In a subset of participants, discrete blood samples were manually drawn every 10 s from 10 to 90 s, every 15 s from 90 s to 3 min, and then at 3.5, 5, 6.5, 8, 13.5, 15, 20, 25, 30, 45, 60, 75, 90, 105, and 120 min. Samples were centrifuged to obtain plasma, and then whole blood and plasma were counted with a calibrated well counter. Radiotracer metabolism was analyzed using plasma samples collected at 3, 8, 15, 30, 60, and 90 min after injection. Metabolites were analyzed using the column-switching high-performance liquid chromatography method (Hilton et al. 2000) to determine the parent fraction.

Regional distribution volume (V_T) was computed using the 1-tissue (1T) compartment model. Each scan and curve were fitted separately. Blood volume in the brain was assumed to be 5% ($C_{\mathrm{PET}}(t) = C_{\mathrm{T}}(t) + 0.05\, C_{\mathrm{B}}(t)$, where $C_{\mathrm{PET}}(t)$, $C_{\mathrm{T}}(t)$, and $C_{\mathrm{B}}(t)$ are the measured PET data, the brain activity, and whole blood activity, respectively).

To compute regional distribution volume relative to the centrum semiovale (DVR_{CS}), we used the simplified reference tissue model with parameter coupling (SRTMC): specifically, all regional time-activity curves were fitted simultaneously per scan with a common parameter k_2', the efflux rate of the pseudo-reference region (coupled parameter fitting).

In addition to V_T and DVR_{CS}, we also evaluated three additional parameters: 1) V_T normalized by the free fraction (f_P), i.e., V_T/f_P, 2) binding potentials with respect to the non-displaceable tracer concentration (BP_{ND}) and 3) to the free tracer concentration (BP_F) (Innis et al. 2007). BP_{ND} and BP_F were calculated indirectly from the V_T estimates using the cerebellum as the reference region, as $(V_{\mathrm{T}} - V_{\mathrm{T}}^{\mathrm{ref}})/V_{\mathrm{T}}^{\mathrm{ref}}$ and BP_F as $(V_{\mathrm{T}} - V_{\mathrm{T}}^{\mathrm{ref}})/f_{\mathrm{P}}$, respectively. BP_F, which is equal to B_{avail}/K_D (with B_{avail} as the concentration of available receptors, and K_D as the receptor dissociation constant) is considered the ideal binding outcome when the metabolite-corrected input function and f_P can be reliably determined.

Cognitive Test Measures:
The CogState SZ Battery consists of a set of computerized cognitive tests to assess speed of processing, attention/vigilance, working memory, visual learning, verbal learning, reasoning/problem solving, and social cognition, domains that are recommended by the MATRICS initiative.
The list of primary outcome measures from the CogState Battery examined in the correlation analysis are:
Detection Task – Reaction Time (log10 transformation)
Identification task – Reaction Time (log10 transformation)
One Card Learning – Accuracy (arcsine transformation)
One Back – Accuracy (arcsine transformation)
Two Back – Accuracy (arcsine transformation)
Groton Maze – Total Errors
International Shopping List – Correct
Social Emotional Cognition – Accuracy (arcsine transformation)

Additional Cognitive Measures:
Rey Auditory Verbal Learning Test (RAVLT) Total recall
RAVLT Interference
RAVLT Short-delay Recall
RAVLT Long-delay Recall
Extra-Dimensional Shift – Accuracy (arcsine transformation)
Intra-Dimensional Shift – Accuracy (arcsine transformation)

Statistical methods:
Group differences between SZ and HC were assessed using a linear mixed-effects model implemented in MATLAB (repeated measures ANCOVA, two-way factorial design). The dependent variable was the regional outcome measure, with Diagnosis (SZ vs HC), Region of Interest (ROI), and their interaction specified as fixed effects. In addition, ROI-wise percent gray matter (%GM) fraction was included as a continuous covariate to account for potential atrophy-related effects. This term was modeled as a fixed linear effect with a shared slope across all subjects.
To account for repeated measurements across regions within individuals, Subject was included as a random intercept. The amygdala served as the reference ROI, and HC served as the reference diagnosis. Models were fit using maximum likelihood estimation. Degrees of freedom and significance testing for fixed effects were obtained using the Satterthwaite approximation. Significance of fixed effects was evaluated using Type III marginal F-tests.
Following a significant diagnosis × ROI interaction, ROI-specific SZ–HC contrasts were extracted from the full interaction model. These contrasts represent group differences within each ROI, conditional on the model. Importantly, these regional effects were estimated within a single unified mixed-effects model, accounting for within-subject correlations and between-subject variability.

Resulting p-values were corrected for multiple comparisons across ROIs using the Benjamini-Hochberg false discovery rate (FDR) procedure (q = 0.05).

Supplementary Results:

Reference region:

Cerebellum ^{11}C-LSN3172176 V_T was lower in SZ than HC (mean ± SD in SZ: 3.85 ± 0.74 mL/cm^3; HC: 4.47 ± 0.66 mL/cm^3; t-test, p=0.04), as was cerebellum %GM (mean ± SD in SZ: 62.51 ± 4.65; HC: 68.79 ± 4.08; t-test, p=0.003). On the other hand, there was no significant group difference in V_T in the CS (2 mL ROI) white matter (WM) (mean ± SD in SZ: 5.76±1.13; HC: 5.54±0.66; t-test, p=0.55), supporting its use as a pseudo-reference region. The pallidum ROI, which is mainly composed of WM according to CAT12 segmentations (%GM < 10%, **Supplementary Table S1**), also did not show significant V_T group differences (mean ± SD in SZ: 13.18±3.43; HC: 13.30±2.19; t-test, p=0.92).

To further evaluate CS as a pseudo-reference region, we extracted CS V_T estimates from paired baseline-scopolamine blocking ^{11}C-LSN3172176 studies performed in n=4 healthy individuals (for details, see (Naganawa et al. 2021)). Mean ± SD of CS V_T were 5.98±0.97 mL/cm^3 for baseline studies and 5.31±0.59 mL/cm^3 for blocking studies, with %differences of -10 ± 14%. The CS V_T values are only moderately higher than the cerebellum V_T values of the same participants (baseline: 5.13±0.56 mL/cm^3; blocking: 5.10±0.51 mL/cm^3). Despite a trend towards a baseline-blocking difference in CS, these results add support to the use of CS as a pseudo-reference region in this work.

Notably, both cerebellum V_T (r=0.57, p=0.003) and CS V_T (r=0.76, p < 0.001) positively correlated with the age of the participants, both in the SZ and HC group (**Supplementary Figure S3**). In future work, we will perform blocking studies in elderly participants (both HC and SZ) to verify if the non-displaceable binding (V_{ND}) increases with age. Additionally, neither cerebellum nor CS V_T correlated with antipsychotic exposure in SZ patients.

Binding potentials relative to the non-displaceable tracer fraction (BP_{ND}), with the cerebellum as the reference region, did not display significant group differences in any region (**Supplementary Table S11A**). On the other hand, binding potentials relative to the free tracer fraction (BP_F) displayed significant group differences, and in very similar regions to V_T and DVR_{CS} (**Supplementary Table S11B**). To better understand this discrepancy, we plotted the inter-parameter similarity across all subjects and ROIs (**Supplementary Figure S5**): while BP_F estimates have higher correlations with the other binding parameters (V_T, r^2=0.95; V_T/f_P, r^2=0.99; DVR_{CS}, r^2=0.92), including DVR_{CS}, BP_{ND} estimates were more weakly correlated with other parameters (V_T, r^2=0.80; V_T/f_P, r^2=0.79, DVR_{CS}, r^2=0.84), probably due to the cerebellum V_T in the denominator amplifying inter-subject variance.

Overall, whether the cerebellum V_T group difference is a confound or a true difference in V_{ND} remains to be fully elucidated.

Gray-matter-masked and partial-volume-corrected data:

Group difference results obtained from GM-masked data and data subjected to PVC are reported in **Supplementary Tables S5** and **S6**, respectively. These results are highly consistent with the main results (reported in **Table 3**), which include %GM as a covariate in the mixed-effects model, with widespread reductions in SZ relative to HC across the same ROIs.

Notably, cerebellum V_T was lower in SZ than HC in non-GM-masked data (mean ± SD in SZ: 3.85 ± 0.74 mL/cm^3; HC: 4.47 ± 0.66 mL/cm^3; t-test, p=0.04), in GM-masked data (mean ± SD in SZ: 4.24 ± 0.85 mL/cm^3; HC: 4.81 ± 0.67 mL/cm^3; t-test, p=0.07), and in PVC data (mean ± SD in SZ: 4.43 ± 0.93 mL/cm^3; HC: 5.11 ± 0.73 mL/cm^3; t-test, p=0.05).

Effect of antipsychotic class on M1 binding:

Antipsychotic class could be a relevant covariate affecting ^{11}C-LSN3172176 binding in SZ patients. However, only 3 patients were currently on first-generation antipsychotics (haloperidol or perphenazine), precluding a meaningful evaluation of class-specific effects. The patients taking first-generation antipsychotics (n=3; haloperidol, perphenazine) did not differ in whole-brain V_T (t-test, p=0.32) and DVR_{CS} (t-test, p=0.83) than those treated with second-generation antipsychotics.

Effect of sex on M1 binding:

Sex could also be a relevant covariate affecting ^{11}C-LSN3172176 binding in HC and/or patients. However, the study was underpowered to answer this question, given the small number of female participants (n=2 out of 16 in both groups). When evaluating the entire cohort (n=4 females, n=28 males), there was no sex difference in whole-brain M1 binding (t-test, DVR_{CS}: p=0.69; V_T: p=0.61), nor in regional M1 binding (all p's > 0.05).

Association between M1 availability and positive symptoms:

We did not detect any significant associations between ^{11}C-LSN3172176 binding and positive symptoms. Prior work has reported an association between positive symptoms and lower muscarinic receptor availability (M1/M4) using ^{123}I-IQNB SPECT in medication-free patients (Raedler et al. 2003). The absence of a correlation in the present study may reflect several factors. First, antipsychotic exposure could attenuate or obscure relationships between receptor availability and current symptom severity, particularly given downstream dopaminergic effects. Second, it remains unclear whether M1 receptor availability primarily reflects a trait-like vulnerability marker rather than a state-dependent correlate of active psychosis, which would weaken cross-sectional associations with positive symptoms. Third, our study primarily includes stable outpatients with schizophrenia. The range of positive symptoms may hence be limited. The efficacy of xanomeline on positive symptoms has been demonstrated in an inpatient sample of schizophrenia (Kaul et al. 2024). A further consideration relates to receptor specificity and binding properties of the radioligands. ^{123}I-IQNB is a non-selective antagonist labeling both M1 and M4 receptors, whereas ^{11}C-LSN3172176 is relatively selective for M1 and, as an agonist radioligand, preferentially binds to the high-affinity (functionally active) receptor state. These differences imply that the two approaches may index distinct receptor pools, with M4, particularly in striatal circuits, likely more directly linked to dopaminergic dysregulation and positive symptoms. This distinction may also help reconcile the apparent discrepancy with xanomeline, a mixed M1/M4 agonist: its antipsychotic effects are thought to be driven predominantly by M4-mediated modulation of striatal dopamine, rather than M1 alone. Thus, a lack of correlation between M1 availability and positive symptoms in the present study does not preclude clinically meaningful effects of dual M1/M4 agonism. Nevertheless, future studies incorporating M4-sensitive ligands or combined circuit-level measures in larger samples may better clarify the relationship between muscarinic signaling and psychotic symptom expression.

Supplementary Figures:

Figure S1. Mean ± SD of metabolite-corrected input function (**A**) and plasma parent fraction (**B**) in schizophrenia (SZ) patients (*n*=11, red) vs. healthy controls (HC) (*n*=14, green). Input functions are reported in semilogarithmic scale.

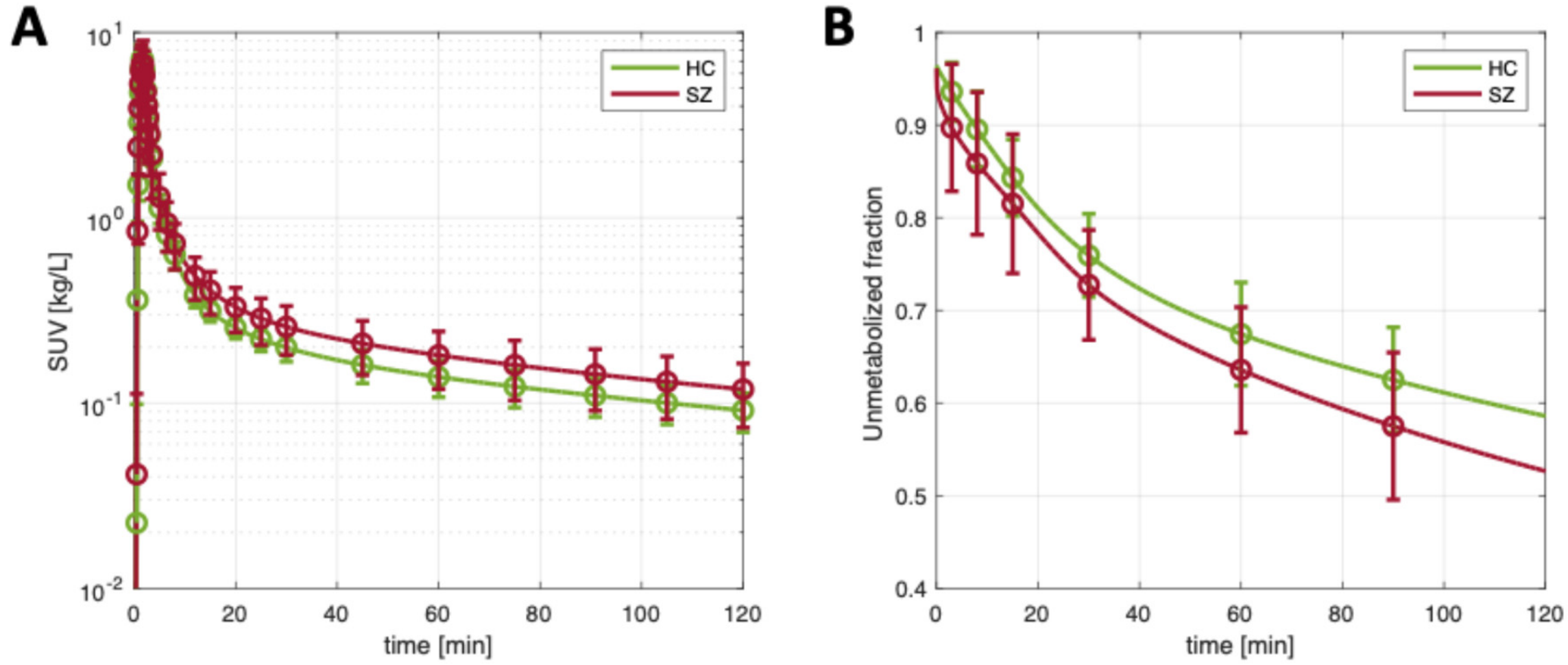

Figure S2. V_T of centrum semiovale (**A**) and cerebellum (**B**) in schizophrenia (SZ) and healthy controls (HC). SZ patients (*n*=11) shown in red, HC participants (A: *n*=16, B, *n*=14), shown in green. The mean of the HC (dashed black line) corresponds to the zero mean difference (Y axis, right, yellow). The mean difference value (corresponding to the mean of the SZ sample, continuous black line) and confidence intervals (vertical error bar) are displayed.

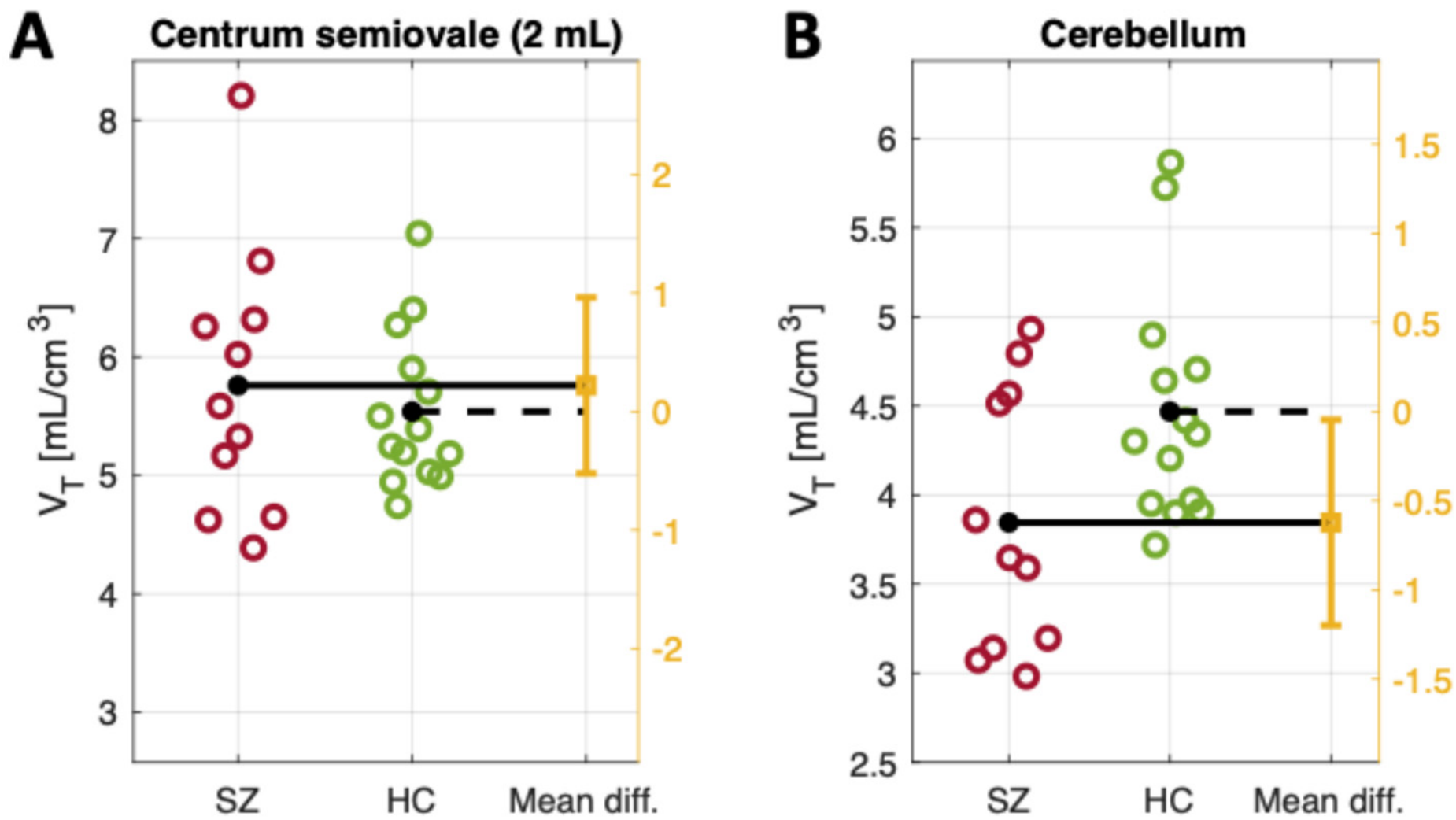

Figure S3. Correlations of age with V_T of centrum semiovale (**A**), cerebellum (**B**) in schizophrenia (SZ) and healthy controls (HC). SZ patients (*n*=11) shown in red, HC participants (A: *n*=16, B, *n*=14), shown in green.

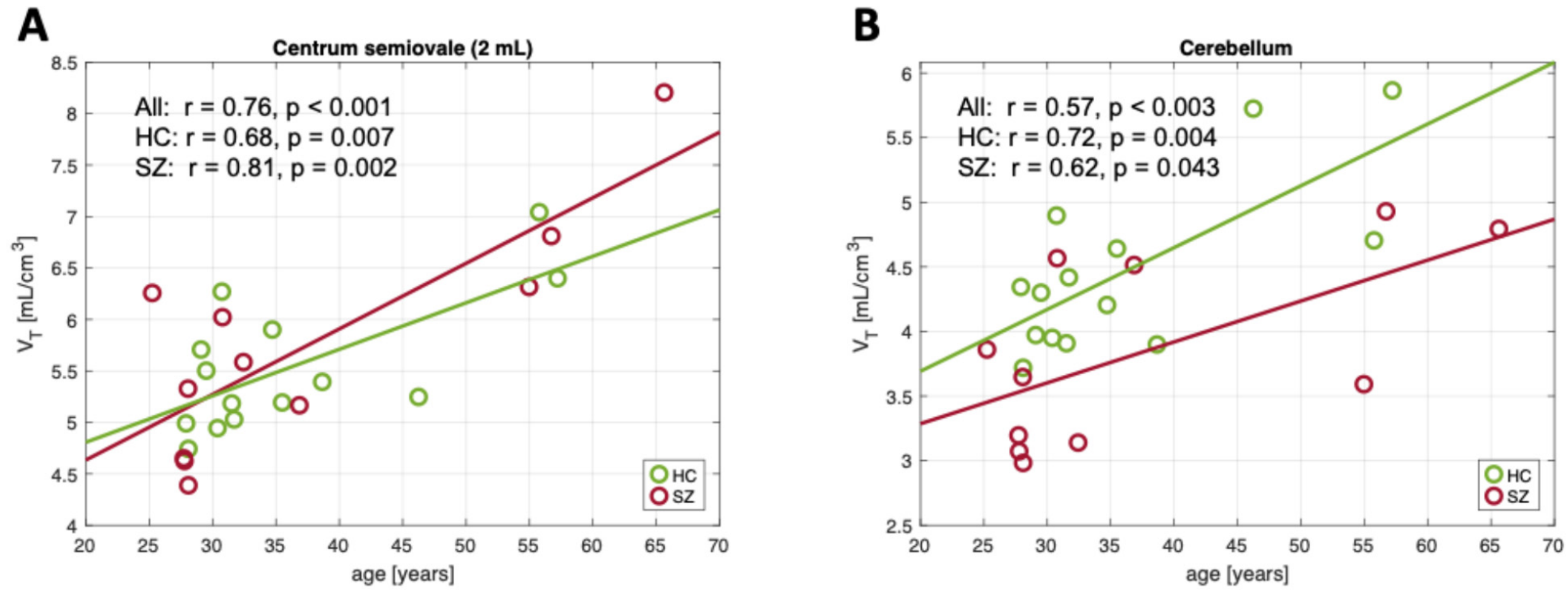

Figure S4. Relationship between whole-brain DVR_{CS} (**A**, n=12), V_T (**B**, n=8), V_T/f_P (**C**, n=8) and antipsychotic dose in CPZ equivalents in schizophrenia.

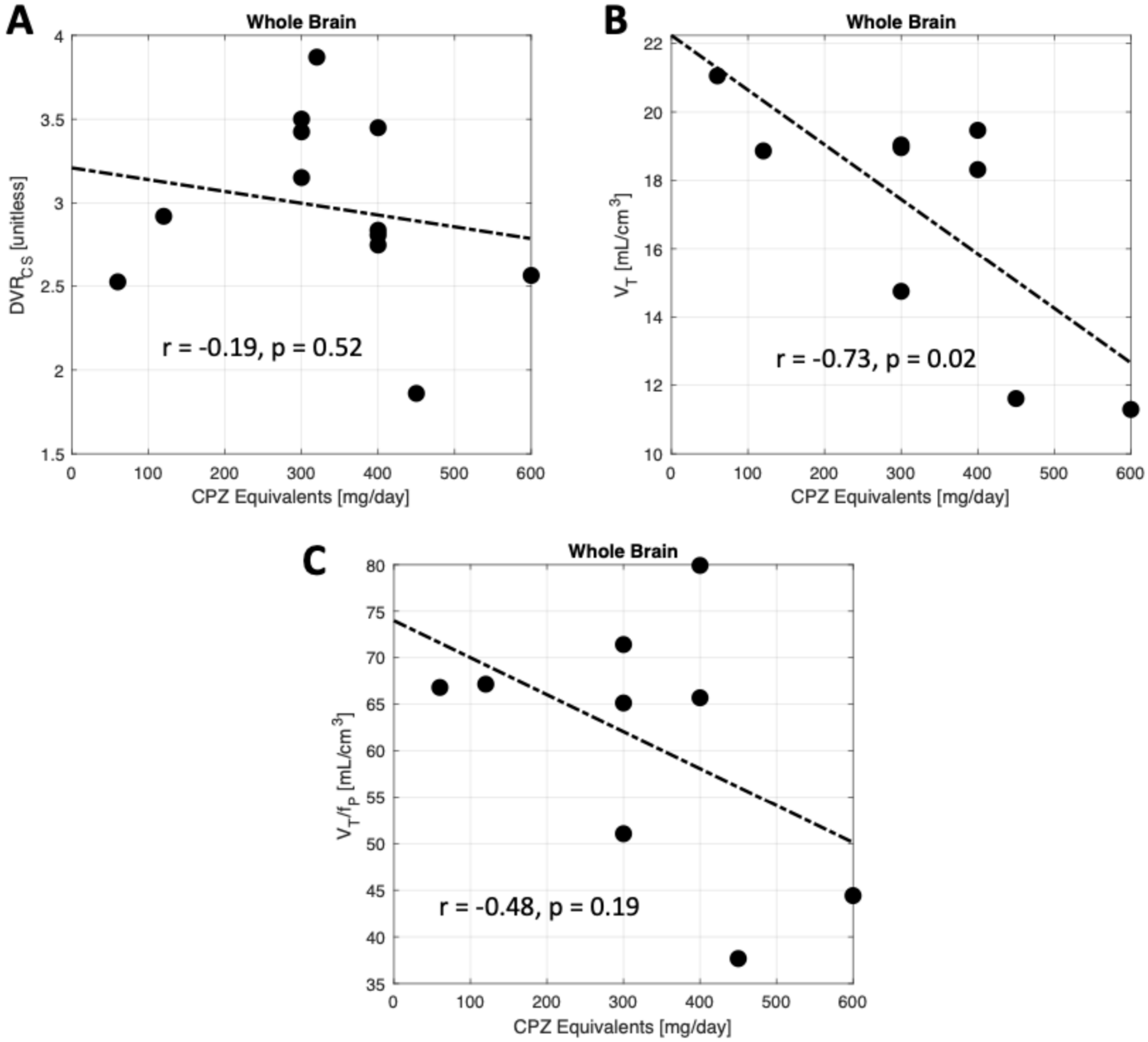

Figure S5. Scatter plots of the similarity between each pair of kinetic parameters reflecting ^{11}C-LSN3172176 binding (V_T, V_T/f_P, BP_F, BP_{ND}, DVR_{CS}), assessed across all ROIs and subjects (n=25, subset of participants with arterial blood samples).

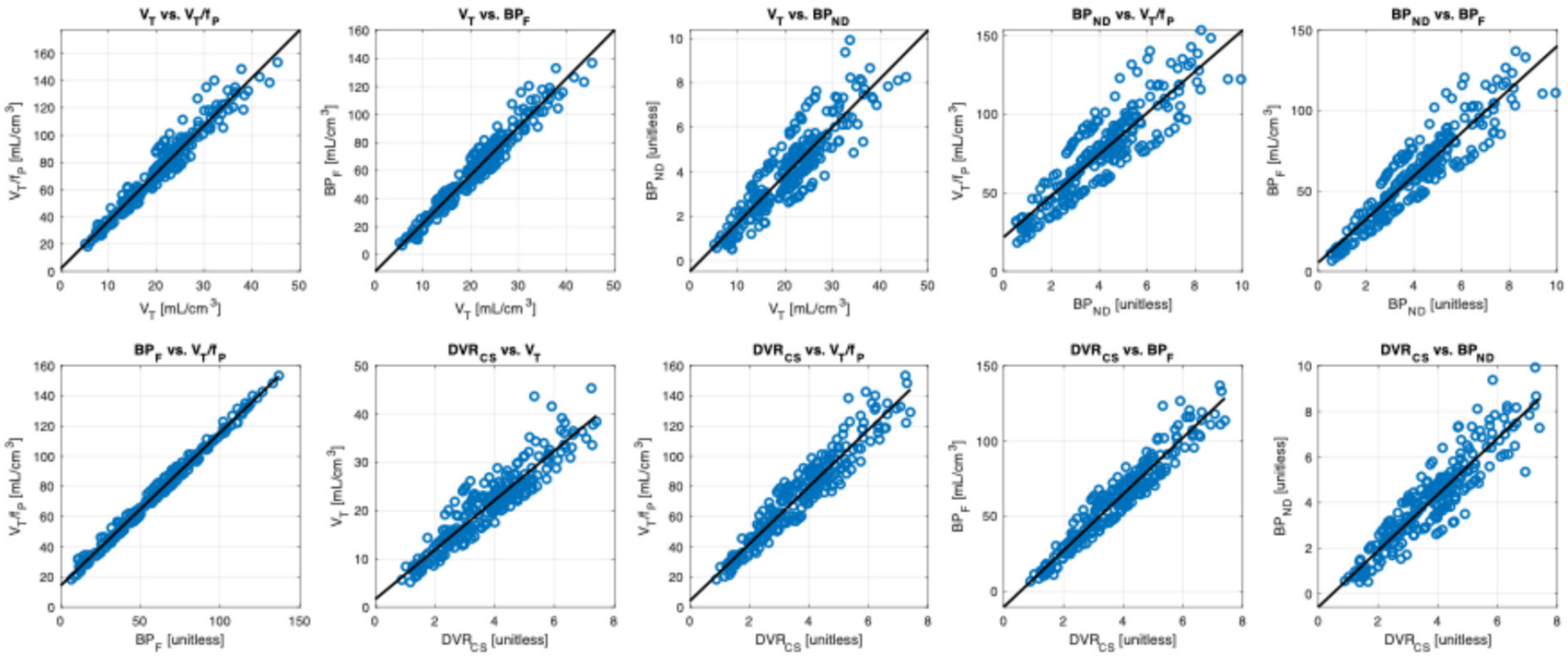

Supplementary Tables:

Table S1. Group differences in ROI-wise percent gray matter (GM) in schizophrenia (SZ) patients vs. healthy controls (HC).

Table S2. Group differences in M1 receptor density ^{11}C-LSN3172176 (DVR_{CS} (A) and V_T (B)) in SZ patients vs. HC, without adjusting for differences in percent gray matter (%GM).

Table S3. Group differences in M1 receptor density ^{11}C-LSN3172176 V_T/f_P in SZ patients vs. HC, adjusted for differences in %GM.

Table S4. Group differences in M1 receptor density ^{11}C-LSN3172176 (DVR_{CS} (A) and V_T (B)) in SZ patients vs. HC, adjusting for differences in %GM and age.

Table S5. Group differences in M1 receptor density ^{11}C-LSN3172176 (DVR_{CS} (A) and V_T (B)) in SZ patients vs. HC, GM-masked data.

Table S6. Group differences in M1 receptor density ^{11}C-LSN3172176 (DVR_{CS} (A) and V_T (B)) in SZ patients vs. HC, partial-volume-corrected data.

Table S7. Group differences in M1 receptor density ^{11}C-LSN3172176 (DVR_{CS} (A) and V_T (B)) in SZ patients vs. HC, adjusted for differences in %GM and smoking status.

Table S8. Group differences in M1 receptor density ^{11}C-LSN3172176 (DVR_{CS} (A) and V_T (B)) in SZ patients vs. HC, adjusted for differences in %GM, excluding SZ patients with Fagerstrom Test for Nicotine Dependence (FTND) > 0.

Table S9. Group differences in M1 receptor density ^{11}C-LSN3172176 (DVR_{CS} (A) and V_T (B)) in SZ patients vs. HC, adjusted for differences in %GM and cannabis use status.

Table S10. Group differences in M1 receptor density ^{11}C-LSN3172176 (DVR_{CS}) in SZ patients vs. HC, adjusted for differences in %GM and current antipsychotic exposure (CPZ equivalent dose x day).

Table S11. Group differences in M1 receptor density ^{11}C-LSN3172176 (BP_{ND} (A), BP_F (B)) in SZ patients vs. HC, adjusting for differences in %GM.

**Table S1.** Group differences in ROI-wise percent gray matter (GM) in schizophrenia (SZ) patients vs. healthy controls (HC)							
		N	**Mean**	**SD**	***p* value (FDR)**	**% diff.**	**Cohen d**
Frontal	HC	16	47.48	4.03	0.195	-6	0.57
	SZ	16	44.83	4.72			
Temporal	HC	16	56.54	2.82	0.064	-8	1.04
	SZ	16	51.80	5.41			
Parietal	HC	16	45.77	3.45	0.195	-6	0.51
	SZ	16	43.10	6.08			
Occipital	HC	16	48.36	3.08	0.081	-8	0.92
	SZ	16	44.58	4.54			
Caudate	HC	16	69.85	9.31	0.067	-6	0.43
	SZ	16	65.78	8.65			
Putamen	HC	16	73.11	5.17	0.242	3	0.48
	SZ	16	75.42	3.74			
Pallidum	HC	16	5.09	2.79	0.195	56	0.83
	SZ	16	7.92	3.58			
Thalamus	HC	16	71.31	8.13	0.067	-6	0.54
	SZ	16	67.22	6.13			
Hippocampus	HC	16	75.53	4.58	0.064	-6	0.71
	SZ	16	71.06	7.11			
Amygdala	HC	16	88.16	4.51	0.305	-2	0.46
	SZ	16	86.24	3.37			
Cerebellum	HC	16	68.79	4.08	0.009*	-9	1.35
	SZ	16	62.51	4.65			

**Table S2A**. Group Differences in M1 receptor availability ^{11}C-LSN3172176 DVR_{CS} in schizophrenia (SZ) patients vs. healthy controls (HC)							
		N	**Mean**	**SD**	***p* value (FDR)**	**% diff.**	**Cohen d**
Frontal	HC	16	4.08	0.35	0.003*	-15	1.21
	SZ	16	3.45	0.62			
Temporal	HC	16	4.60	0.44	<0.001*	-19	1.45
	SZ	16	3.73	0.69			
Parietal	HC	16	4.06	0.43	0.002*	-16	1.15
	SZ	16	3.41	0.65			
Occipital	HC	16	4.31	0.49	<0.001*	-19	1.50
	SZ	16	3.49	0.58			
Caudate	HC	16	5.34	0.95	<0.001*	-22	1.12
	SZ	16	4.18	1.08			
Putamen	HC	16	6.51	0.56	<0.001*	-17	1.32
	SZ	16	5.39	1.03			
Pallidum	HC	16	2.42	0.33	0.698	-3	0.24
	SZ	16	2.35	0.29			
Thalamus	HC	16	1.60	0.13	0.401	-12	1.17
	SZ	16	1.40	0.19			
Hippocampus	HC	16	3.04	0.38	0.006*	-19	1.29
	SZ	16	2.47	0.48			
Amygdala	HC	16	4.62	0.57	<0.001*	-21	1.33
	SZ	16	3.66	0.81			
Cerebellum	HC	16	0.85	0.10	0.679	-12	0.97
	SZ	16	0.75	0.10			

**Table S2B.** Group Differences in M1 receptor availability ^{11}C-LSN3172176 V_T in schizophrenia (SZ) patients vs. healthy controls (HC)							
Frontal	HC	14	22.17	1.83	0.085	-11	0.86
	SZ	11	19.68	3.71			
Temporal	HC	14	25.28	2.26	0.007*	-16	1.19
	SZ	11	21.32	4.12			
Parietal	HC	14	22.27	2.23	0.054	-13	1.00
	SZ	11	19.37	3.40			
Occipital	HC	14	23.70	2.73	0.007*	-17	1.21
	SZ	11	19.72	3.69			
Caudate	HC	14	29.06	4.48	0.002*	-16	0.83
	SZ	11	24.30	6.71			
Putamen	HC	14	36.30	3.88	<0.001*	-15	0.85
	SZ	11	31.03	7.93			
Pallidum	HC	14	13.30	2.19	0.930	-1	0.04
	SZ	11	13.18	3.43			
Thalamus	HC	14	8.52	0.87	0.404	-15	1.22
	SZ	11	7.22	1.20			
Hippocampus	HC	14	16.34	1.53	0.085	-16	1.19
	SZ	11	13.77	2.64			
Amygdala	HC	14	25.59	2.61	0.003*	-17	1.12

	SZ	11	21.16	4.96			
Cerebellum	HC	14	4.47	0.66	0.705	-14	0.87
	SZ	11	3.85	0.74			

**Table S3.** Group differences in M1 receptor availability ^{11}C-LSN3172176 V_T/f_P in schizophrenia (SZ) patients vs. healthy controls (HC) (adjusted for differences in percent gray matter (GM))							
		N	**Mean**	**SD**	***p* value (FDR)**	**% diff.**	**Cohen d**
Frontal	HC	14	80.77	5.93	0.023*	-13	1.04
	SZ	11	69.66	13.30			
Temporal	HC	14	92.07	7.15	0.003*	-15	1.43
	SZ	11	75.39	14.21			
Parietal	HC	14	81.20	8.00	0.014*	-14	1.11
	SZ	11	68.64	12.96			
Occipital	HC	14	86.31	8.62	0.003*	-16	1.45
	SZ	11	69.70	12.70			
Caudate	HC	14	105.92	15.37	<0.001*	-18	0.98
	SZ	11	85.96	22.40			
Putamen	HC	14	132.00	10.23	<0.001*	-19	1.14
	SZ	11	109.36	25.31			
Pallidum	HC	14	48.21	5.97	0.235	-11	0.23
	SZ	11	46.27	9.96			
Thalamus	HC	14	30.97	2.26	0.693	-5	1.54
	SZ	11	25.53	4.25			
Hippocampus	HC	14	59.49	4.29	0.076	-13	1.39
	SZ	11	48.81	9.62			
Amygdala	HC	14	93.18	8.13	<0.001*	-18	1.33
	SZ	11	74.81	16.97			
Cerebellum	HC	14	16.28	2.31	0.592	16	1.03
	SZ	11	13.58	2.58			

**Table S4A**. Group Differences in M1 receptor availability<br>^{11}C-LSN3172176 DVR_{CS} in schizophrenia (SZ) patients vs. healthy controls (HC)<br>(adjusted for differences in percent gray matter (GM) and age)							
		N	**Mean**	**SD**	***p* value (FDR)**	**% diff.**	**Cohen d**
Frontal	HC	16	4.08	0.35	0.005*	-12	1.21
	SZ	16	3.45	0.62			
Temporal	HC	16	4.60	0.44	<0.001*	-14	1.45
	SZ	16	3.73	0.69			
Parietal	HC	16	4.06	0.43	0.004*	-13	1.15
	SZ	16	3.41	0.65			
Occipital	HC	16	4.31	0.49	<0.001*	-15	1.50
	SZ	16	3.49	0.58			
Caudate	HC	16	5.34	0.95	<0.001*	-18	1.12
	SZ	16	4.18	1.08			
Putamen	HC	16	6.51	0.56	<0.001*	-18	1.32
	SZ	16	5.39	1.03			
Pallidum	HC	16	2.42	0.33	0.405	-6	0.24
	SZ	16	2.35	0.29			
Thalamus	HC	16	1.60	0.13	0.924	-1	1.17
	SZ	16	1.40	0.19			
Hippocampus	HC	16	3.04	0.38	0.039*	-12	1.29
	SZ	16	2.47	0.48			
Amygdala	HC	16	4.62	0.57	<0.001*	-19	1.33
	SZ	16	3.66	0.81			
Cerebellum	HC	16	0.85	0.10	0.405	19	0.97
	SZ	16	0.75	0.10			

**Table S4B.** Group Differences in M1 receptor availability<br>^{11}C-LSN3172176 V_T in schizophrenia (SZ) patients vs. healthy controls (HC)<br>(adjusted for differences in percent gray matter (GM) and age)							
		N	**Mean**	**SD**	***p* value (FDR)**	**% diff.**	**Cohen d**
Frontal	HC	14	22.17	1.83	0.088	-10	0.86
	SZ	11	19.68	3.71			
Temporal	HC	14	25.28	2.26	0.025*	-12	1.19
	SZ	11	21.32	4.12			
Parietal	HC	14	22.27	2.23	0.064	-11	1.00
	SZ	11	19.37	3.40			
Occipital	HC	14	23.70	2.73	0.021*	-14	1.21
	SZ	11	19.72	3.69			
Caudate	HC	14	29.06	4.48	0.001*	-16	0.83
	SZ	11	24.30	6.71			
Putamen	HC	14	36.30	3.88	<0.001*	-17	0.85
	SZ	11	31.03	7.93			
Pallidum	HC	14	13.30	2.19	0.364	-9	0.04
	SZ	11	13.18	3.43			
Thalamus	HC	14	8.52	0.87	0.832	-3	1.22
	SZ	11	7.22	1.20			

Hippocampus	HC	14	16.34	1.53	0.170	-11	1.19
	SZ	11	13.77	2.64			
Amygdala	HC	14	25.59	2.61	0.003*	-16	1.12
	SZ	11	21.16	4.96			
Cerebellum	HC	14	4.47	0.66	0.526	19	0.87
	SZ	11	3.85	0.74			

**Table S5A**. Group Differences in M1 receptor availability ^{11}C-LSN3172176 DVR_{CS} in schizophrenia (SZ) patients vs. healthy controls (HC), GM-masked data							
		N	**Mean**	**SD**	***p* value (FDR)**	**% diff.**	**Cohen d**
Frontal	HC	16	33.6	2.47	0.074	-11	0.87
	SZ	16	29.88	5.61			
Temporal	HC	16	35.17	3.04	0.024*	-14	1.07
	SZ	16	30.26	5.77			
Parietal	HC	16	34.65	3.21	0.037*	-12	1.00
	SZ	16	30.31	5.24			
Occipital	HC	16	35.25	3.56	0.013*	-15	1.15
	SZ	16	29.77	5.7			
Caudate	HC	16	39.59	3.92	0.001*	-18	1.07
	SZ	16	32.53	8.58			
Putamen	HC	16	46.95	4.72	<0.001*	-18	1.09
	SZ	16	38.4	10.19			
Pallidum	HC	16	25.66	8.32	0.460	-6	0.19
	SZ	16	24.14	6.79			
Thalamus	HC	16	9.57	1.09	0.460	-16	1.27
	SZ	16	8.00	1.32			
Hippocampus	HC	16	19.51	1.89	0.159	-15	1.19
	SZ	16	16.56	2.91			
Amygdala	HC	16	28.23	2.56	0.030*	-16	1.08
	SZ	16	23.61	5.57			
Cerebellum	HC	16	0.91	0.11	0.784	-19	0.2
	SZ	16	0.82	0.10			

**Table S5B.** Group Differences in M1 receptor availability ^{11}C-LSN3172176 V_T in schizophrenia (SZ) patients vs. healthy controls (HC), GM-masked data							
Frontal	HC	14	26.37	1.71	0.093	-11	0.89
	SZ	11	23.34	4.61			
Temporal	HC	14	28.75	2.44	0.013*	-15	1.15
	SZ	11	24.33	4.92			
Parietal	HC	14	26.38	2.50	0.058	-13	0.99
	SZ	11	22.93	4.24			
Occipital	HC	14	27.85	2.85	0.009*	-17	1.30
	SZ	11	23.00	4.38			
Caudate	HC	14	35.16	3.42	0.001*	-18	1.08
	SZ	11	28.88	7.62			
Putamen	HC	14	41.89	4.17	<0.001*	-18	1.12
	SZ	11	34.21	8.83			
Pallidum	HC	14	19.76	7.35	0.854	-1	0.04
	SZ	11	19.47	5.51			
Thalamus	HC	14	8.88	0.94	0.458	-16	1.29
	SZ	11	7.46	1.21			
Hippocampus	HC	14	17.43	1.62	0.144	-15	1.18
	SZ	11	14.82	2.66			
Amygdala	HC	14	26.81	2.54	0.011*	-17	1.13

	SZ	11	22.18	5.24			
Cerebellum	HC	14	4.81	0.67	0.792	-12	0.74
	SZ	11	4.24	0.85			

**Table S6A**. Group Differences in M1 receptor availability ^{11}C-LSN3172176 DVR_{CS} in schizophrenia (SZ) patients vs. healthy controls (HC), partial-volume-corrected data							
		N	**Mean**	**SD**	***p* value (FDR)**	**% diff.**	**Cohen d**
Frontal	HC	16	6.26	0.57	0.006*	-14	1.05
	SZ	16	5.37	1.00			
Temporal	HC	16	6.56	0.67	<0.001*	-17	1.27
	SZ	16	5.44	1.02			
Parietal	HC	16	6.43	0.67	0.003*	-15	1.02
	SZ	16	5.49	1.08			
Occipital	HC	16	6.54	0.72	<0.001*	-17	1.26
	SZ	16	5.4	1.01			
Caudate	HC	16	7.43	0.92	<0.001*	-22	1.41
	SZ	16	5.79	1.32			
Putamen	HC	16	8.68	0.73	<0.001*	-21	1.56
	SZ	16	6.84	1.45			
Pallidum	HC	16	4.7	1.56	0.534	-4	0.15
	SZ	16	4.49	1.21			
Thalamus	HC	16	1.76	0.17	0.534	-13	1.09
	SZ	16	1.53	0.23			
Hippocampus	HC	16	3.67	0.47	0.046*	-18	1.19
	SZ	16	3.03	0.58			
Amygdala	HC	16	5.32	0.72	<0.001*	-21	1.25
	SZ	16	4.22	0.98			
Cerebellum	HC	16	0.95	0.12	0.729	-11	0.19
	SZ	16	0.84	0.13			

**Table S6B.** Group Differences in M1 receptor availability ^{11}C-LSN3172176 V_T in schizophrenia (SZ) patients vs. healthy controls (HC), partial-volume-corrected data							
Frontal	HC	14	33.6	2.47	0.074	-11	0.87
	SZ	11	29.88	5.61			
Temporal	HC	14	35.17	3.04	0.024*	-14	1.07
	SZ	11	30.26	5.77			
Parietal	HC	14	34.65	3.21	0.037*	-12	1.00
	SZ	11	30.31	5.24			
Occipital	HC	14	35.25	3.56	0.013*	-15	1.15
	SZ	11	29.77	5.7			
Caudate	HC	14	39.59	3.92	0.001*	-18	1.07
	SZ	11	32.53	8.58			
Putamen	HC	14	46.95	4.72	<0.001*	-18	1.09
	SZ	11	38.4	10.19			
Pallidum	HC	14	25.66	8.32	0.460	-6	0.19
	SZ	11	24.14	6.79			
Thalamus	HC	14	9.57	1.09	0.460	-16	1.27
	SZ	11	8.00	1.32			
Hippocampus	HC	14	19.51	1.89	0.159	-15	1.19
	SZ	11	16.56	2.91			
Amygdala	HC	14	28.23	2.56	0.030*	-16	1.08

	SZ	11	23.61	5.57			
Cerebellum	HC	14	5.12	0.73	0.712	-14	0.82
	SZ	11	4.43	0.93			

**Table S7A**. Group Differences in M1 receptor availability ^{11}C-LSN3172176 *DVR*CS in schizophrenia (SZ) patients vs. healthy controls (HC) (adjusted for differences in percent gray matter (GM) and smoking status)							
		N	**Mean**	**SD**	***p* value (FDR)**	**% diff.**	**Cohen d**
Frontal	HC	16	4.08	0.35	0.042*	-10	1.21
	SZ	16	3.45	0.62			
Temporal	HC	16	4.60	0.44	0.005*	-13	1.45
	SZ	16	3.73	0.69			
Parietal	HC	16	4.06	0.43	0.036*	-11	1.15
	SZ	16	3.41	0.65			
Occipital	HC	16	4.31	0.49	0.005*	-13	1.50
	SZ	16	3.49	0.58			
Caudate	HC	16	5.34	0.95	<0.001*	-17	1.12
	SZ	16	4.18	1.08			
Putamen	HC	16	6.51	0.56	<0.001*	-17	1.32
	SZ	16	5.39	1.03			
Pallidum	HC	16	2.42	0.33	0.719	-3	0.24
	SZ	16	2.35	0.29			
Thalamus	HC	16	1.60	0.13	0.719	4	1.17
	SZ	16	1.40	0.19			
Hippocampus	HC	16	3.04	0.38	0.164	-10	1.29
	SZ	16	2.47	0.48			
Amygdala	HC	16	4.62	0.57	<0.001*	-17	1.33
	SZ	16	3.66	0.81			
Cerebellum	HC	16	0.85	0.10	0.240	29	0.97
	SZ	16	0.75	0.10			

**Table S7B.** Group Differences in M1 receptor availability ^{11}C-LSN3172176 V_T in schizophrenia (SZ) patients vs. healthy controls (HC) (adjusted for differences in percent gray matter (GM) and smoking status)							
		N	**Mean**	**SD**	***p* value (FDR)**	**% diff.**	**Cohen d**
Frontal	HC	14	22.17	1.83	0.386	-6	0.86
	SZ	11	19.68	3.71			
Temporal	HC	14	25.28	2.26	0.212	-9	1.19
	SZ	11	21.32	4.12			
Parietal	HC	14	22.27	2.23	0.323	-8	1.00
	SZ	11	19.37	3.40			
Occipital	HC	14	23.70	2.73	0.203	-10	1.21
	SZ	11	19.72	3.69			
Caudate	HC	14	29.06	4.48	0.033*	-13	0.83
	SZ	11	24.30	6.71			
Putamen	HC	14	36.30	3.88	0.001*	-14	0.85
	SZ	11	31.03	7.93			
Pallidum	HC	14	13.30	2.19	0.798	-3	0.04
	SZ	11	13.18	3.43			
Thalamus	HC	14	8.52	0.87	0.726	7	1.22
	SZ	11	7.22	1.20			

Hippocampus	HC	14	16.34	1.53	0.548	-6	1.19
	SZ	11	13.77	2.64			
Amygdala	HC	14	25.59	2.61	0.056	-13	1.12
	SZ	11	21.16	4.96			
Cerebellum	HC	14	4.47	0.66	0.323	38	0.87
	SZ	11	3.85	0.74			

**Table S8A**. Group Differences in M1 receptor availability<br>^{11}C-LSN3172176 DVR_{CS} in schizophrenia (SZ) patients vs. healthy controls (HC)<br>(adjusted for differences in percent gray matter (GM), excluding patients with FTND > 0)							
		N	**Mean**	**SD**	***p* value (FDR)**	**% diff.**	**Cohen d**
Frontal	HC	16	4.08	0.35	0.032*	-10	1.11
	SZ	14	3.61	0.49			
Temporal	HC	16	4.60	0.44	<0.003*	-12	1.36
	SZ	14	3.89	0.58			
Parietal	HC	16	4.06	0.43	0.025*	-10	1.01
	SZ	14	3.57	0.52			
Occipital	HC	16	4.31	0.49	0.002*	-13	1.40
	SZ	14	3.62	0.48			
Caudate	HC	16	5.34	0.95	<0.001*	-16	0.98
	SZ	14	4.43	0.85			
Putamen	HC	16	6.51	0.56	<0.001*	-16	1.27
	SZ	16	5.57	0.87			
Pallidum	HC	16	2.42	0.33	0.567	-5	0.10
	SZ	16	2.39	0.25			
Thalamus	HC	16	1.60	0.13	0.924	-1	1.08
	SZ	14	1.46	0.12			
Hippocampus	HC	16	3.04	0.38	0.056	-11	1.18
	SZ	14	2.60	0.25			
Amygdala	HC	16	4.62	0.57	0.001*	-16	1.22
	SZ	14	3.84	0.69			
Cerebellum	HC	16	0.85	0.10	0.473	17	0.82
	SZ	14	0.77	0.09			

**Table S8B.** Group Differences in M1 receptor availability<br>^{11}C-LSN3172176 V_T in schizophrenia (SZ) patients vs. healthy controls (HC)<br>(adjusted for differences in percent gray matter (GM), excluding patients with FTND > 0)							
Frontal	HC	14	22.17	1.83	0.123	-9	0.77
	SZ	9	19.68	3.71			
Temporal	HC	14	25.28	2.26	0.015*	-12	1.16
	SZ	9	21.32	4.12			
Parietal	HC	14	22.27	2.23	0.059	-10	0.86
	SZ	9	19.37	3.40			
Occipital	HC	14	23.70	2.73	0.007*	-14	1.25
	SZ	9	19.72	3.69			
Caudate	HC	14	29.06	4.48	0.008*	-14	0.68
	SZ	9	24.30	6.71			
Putamen	HC	14	36.30	3.88	<0.001*	-17	1.03
	SZ	9	31.03	7.93			
Pallidum	HC	14	13.30	2.19	0.345	-10	0.19
	SZ	9	13.18	3.43			
Thalamus	HC	14	8.52	0.87	0.680	-6	1.22
	SZ	9	7.22	1.20			
Hippocampus	HC	14	16.34	1.53	0.123	-13	1.08

	SZ	9	13.77	2.64			
Amygdala	HC	14	25.59	2.61	0.008*	-14	1.10
	SZ	9	21.16	4.96			
Cerebellum	HC	14	4.47	0.66	0.613	16	0.98
	SZ	9	3.85	0.74			

**Table S9A**. Group Differences in M1 receptor availability ^{11}C-LSN3172176 *DVR*$_{CS}$ in schizophrenia (SZ) patients vs. healthy controls (HC) adjusted for differences in percent gray matter (GM) and cannabis use status)							
		N	**Mean**	**SD**	***p* value (FDR)**	**% diff.**	**Cohen d**
Frontal	HC	16	4.08	0.35	0.010*	-13	1.21
	SZ	16	3.45	0.62			
Temporal	HC	16	4.60	0.44	0.001*	-15	1.45
	SZ	16	3.73	0.69			
Parietal	HC	16	4.06	0.43	0.008*	-13	1.15
	SZ	16	3.41	0.65			
Occipital	HC	16	4.31	0.49	0.001*	-16	1.50
	SZ	16	3.49	0.58			
Caudate	HC	16	5.34	0.95	<0.001*	-19	1.12
	SZ	16	4.18	1.08			
Putamen	HC	16	6.51	0.56	<0.001*	-18	1.32
	SZ	16	5.39	1.03			
Pallidum	HC	16	2.42	0.33	0.425	-7	0.24
	SZ	16	2.35	0.29			
Thalamus	HC	16	1.60	0.13	0.861	-2	1.17
	SZ	16	1.40	0.19			
Hippocampus	HC	16	3.04	0.38	0.054	-13	1.29
	SZ	16	2.47	0.48			
Amygdala	HC	16	4.62	0.57	<0.001*	-19	1.33
	SZ	16	3.66	0.81			
Cerebellum	HC	16	0.85	0.10	0.499	17	0.97
	SZ	16	0.75	0.10			

**Table S9B.** Group Differences in M1 receptor availability ^{11}C-LSN3172176 V_T in schizophrenia (SZ) patients vs. healthy controls (HC) (adjusted for differences in percent gray matter (GM) and cannabis use status)							
		N	**Mean**	**SD**	***p* value (FDR)**	**% diff.**	**Cohen d**
Frontal	HC	14	22.17	1.83	0.324	-7	0.86
	SZ	11	19.68	3.71			
Temporal	HC	14	25.28	2.26	0.165	-9	1.19
	SZ	11	21.32	4.12			
Parietal	HC	14	22.27	2.23	0.308	-8	1.00
	SZ	11	19.37	3.40			
Occipital	HC	14	23.70	2.73	0.154	-10	1.21
	SZ	11	19.72	3.69			
Caudate	HC	14	29.06	4.48	0.019*	-13	0.83
	SZ	11	24.30	6.71			
Putamen	HC	14	36.30	3.88	<0.001*	-15	0.85
	SZ	11	31.03	7.93			
Pallidum	HC	14	13.30	2.19	0.725	-3	0.04
	SZ	11	13.18	3.43			
Thalamus	HC	14	8.52	0.87	0.725	6	1.22
	SZ	11	7.22	1.20			

Hippocampus	HC	14	16.34	1.53	0.485	-7	1.19
	SZ	11	13.77	2.64			
Amygdala	HC	14	25.59	2.61	0.037*	-13	1.12
	SZ	11	21.16	4.96			
Cerebellum	HC	14	4.47	0.66	0.324	36	0.87
	SZ	11	3.85	0.74			

**Table S10**. Group Differences in M1 receptor availability<br>^{11}C-LSN3172176 *DVR*$_{CS}$ in schizophrenia (SZ) patients vs. healthy controls (HC)<br>adjusted for differences in percent gray matter (GM) and antipsychotic exposure)							
		N	**Mean**	**SD**	***p* value (FDR)**	**% diff.**	**Cohen d**
Frontal	HC	16	4.08	0.35	0.446	-5	1.21
	SZ	16	3.45	0.62			
Temporal	HC	16	4.60	0.44	0.183	-8	1.45
	SZ	16	3.73	0.69			
Parietal	HC	16	4.06	0.43	0.432	-5	1.15
	SZ	16	3.41	0.65			
Occipital	HC	16	4.31	0.49	0.183	-8	1.50
	SZ	16	3.49	0.58			
Caudate	HC	16	5.34	0.95	0.001*	-13	1.12
	SZ	16	4.18	1.08			
Putamen	HC	16	6.51	0.56	<0.001*	-13	1.32
	SZ	16	5.39	1.03			
Pallidum	HC	16	2.42	0.33	0.521	6	0.24
	SZ	16	2.35	0.29			
Thalamus	HC	16	1.60	0.13	0.248	19	1.17
	SZ	16	1.40	0.19			
Hippocampus	HC	16	3.04	0.38	0.759	-2	1.29
	SZ	16	2.47	0.48			
Amygdala	HC	16	4.62	0.57	0.031*	-12	1.33
	SZ	16	3.66	0.81			
Cerebellum	HC	16	0.85	0.10	0.067	56	0.97
	SZ	16	0.75	0.10			

**Table S11A**. Group Differences in M1 receptor availability ^{11}C-LSN3172176 BP_{ND} in schizophrenia (SZ) patients vs. healthy controls (HC) (adjusted for differences in percent gray matter (GM))							
		N	**Mean**	**SD**	***p* value (FDR)**	**% diff.**	**Cohen d**
Frontal	HC	14	4.04	0.66	0.874	6	-0.18
	SZ	11	4.20	1.03			
Temporal	HC	14	4.74	0.79	0.874	3	0.11
	SZ	11	4.64	1.18			
Parietal	HC	14	4.06	0.72	0.874	5	-0.08
	SZ	11	4.13	1.08			
Occipital	HC	14	4.39	0.82	0.954	0	0.21
	SZ	11	4.20	0.94			
Caudate	HC	14	5.63	1.32	0.874	-2	0.13
	SZ	11	5.42	1.80			
Putamen	HC	14	7.24	1.14	0.874	-5	0.09
	SZ	11	7.10	1.75			
Pallidum	HC	14	2.01	0.51	0.874	7	-0.81
	SZ	11	2.41	0.45			
Thalamus	HC	14	0.93	0.20	0.874	28	0.12
	SZ	11	0.90	0.26			
Hippocampus	HC	14	2.72	0.56	0.874	5	0.08
	SZ	11	2.66	0.86			
Amygdala	HC	14	4.82	0.86	0.876	-2	0.16
	SZ	11	4.62	1.59			

**Table S11B.** Group Differences in M1 receptor availability ^{11}C-LSN3172176 BP_{F} in schizophrenia (SZ) patients vs. healthy controls (HC) (adjusted for differences in percent gray matter (GM))							
Frontal	HC	14	64.49	6.00	0.090	-12	0.86
	SZ	11	56.07	12.56			
Temporal	HC	14	75.79	7.42	0.018*	-14	1.19
	SZ	11	61.81	13.71			
Parietal	HC	14	64.91	8.19	0.064	-13	1.00
	SZ	11	55.06	12.36			
Occipital	HC	14	70.02	9.12	0.016*	-16	1.21
	SZ	11	56.12	11.81			
Caudate	HC	14	89.63	16.31	<0.001*	-18	0.83
	SZ	11	72.38	22.57			
Putamen	HC	14	115.72	10.65	<0.001*	-20	0.85
	SZ	11	95.78	24.95			
Pallidum	HC	14	31.93	6.30	0.510	-9	0.04
	SZ	11	32.69	8.46			
Thalamus	HC	14	14.69	2.21	0.752	9	1.22
	SZ	11	11.94	2.91			
Hippocampus	HC	14	43.20	5.14	0.249	-12	1.19
	SZ	11	35.23	9.22			
Amygdala	HC	14	76.90	8.50	0.001*	-18	1.12

	SZ	11	61.23	17.17			

References

Hilton, J., F. Yokoi, R. F. Dannals, H. T. Ravert, Z. Szabo, and D. F. Wong. 2000. 'Column-switching HPLC for the analysis of plasma in PET imaging studies', *Nucl Med Biol*, 27: 627-30.

Innis, Robert B, Vincent J Cunningham, Jacques Delforge, Masahiro Fujita, Albert Gjedde, Roger N Gunn, James Holden, Sylvain Houle, Sung-Cheng Huang, Masanori Ichise, Hidehiro Iida, Hiroshi Ito, Yuichi Kimura, Robert A Koeppe, Gitte M Knudsen, Juhani Knuuti, Adriaan A Lammertsma, Marc Laruelle, Jean Logan, Ralph Paul Maguire, Mark A Mintun, Evan D Morris, Ramin Parsey, Julie C Price, Mark Slifstein, Vesna Sossi, Tetsuya Suhara, John R Votaw, Dean F Wong, and Richard E Carson. 2007. 'Consensus Nomenclature for in vivo Imaging of Reversibly Binding Radioligands', *Journal of Cerebral Blood Flow & Metabolism*, 27: 1533-39.

Kaul, Inder, Sharon Sawchak, David P. Walling, Carol A. Tamminga, Alan Breier, Haiyuan Zhu, Andrew C. Miller, Steven M. Paul, and Stephen K. Brannan. 2024. 'Efficacy and Safety of Xanomeline-Trospium Chloride in Schizophrenia: A Randomized Clinical Trial', *JAMA Psychiatry*, 81: 749-56.

Naganawa, M., N. Nabulsi, S. Henry, D. Matuskey, S. F. Lin, L. Slieker, A. J. Schwarz, N. Kant, C. Jesudason, K. Ruley, A. Navarro, H. Gao, J. Ropchan, D. Labaree, R. E. Carson, and Y. Huang. 2021. 'First-in-Human Assessment of (11)C-LSN3172176, an M1 Muscarinic Acetylcholine Receptor PET Radiotracer', *J Nucl Med*, 62: 553-60.

Raedler, T. J., M. B. Knable, D. W. Jones, R. A. Urbina, J. G. Gorey, K. S. Lee, M. F. Egan, R. Coppola, and D. R. Weinberger. 2003. 'In vivo determination of muscarinic acetylcholine receptor availability in schizophrenia', *Am J Psychiatry*, 160: 118-27.
